# Self-oxidatively crosslinked sprayable hydrogel for microenvironment remodeling and accelerated healing of recurrent aphthous ulcers

**DOI:** 10.1016/j.mtbio.2026.103240

**Published:** 2026-05-14

**Authors:** Lihong Zhou, Jingyu Yan, Yurong Xu, Chenying Cui, Kaifang Zhang, Kun Liu, Xiuping Wu, Bing Li

**Affiliations:** a Shanxi Medical University School and Hospital of Stomatology, Taiyuan, Shanxi, 030001, China; b Shanxi Province Key Laboratory of Oral Diseases Prevention and New Materials, Taiyuan, Shanxi, 030001, China

**Keywords:** Recurrent aphthous ulcer (RAU), Self-oxidatively crosslinked hydrogel, Immunomodulation, Macrophage polarization, Reactive oxygen species (ROS) scavenging, Wound healing

## Abstract

Recurrent aphthous ulcer (RAU) is a prevalent inflammatory disorder of the oral mucosa, characterized by a persistent “vicious cycle” of excessive oxidative stress, dysregulated immune responses, and impaired tissue regeneration. Effective clinical management remains challenging due to the dynamic and hydrated oral environment, which severely limits drug retention and multi-target therapeutic efficacy. Herein, we developed a multifunctional, sprayable hydrogel (PDS Gel) using a hierarchical “oxygen-limited initiation – oxygen-controlled progression” crosslinking strategy, integrating polygalacturonic acid (PGA), dopamine (DA), and salidroside-derived carbon dots (Sal-CDs). The PDS Gel achieves rapid in situ solidification and exhibits certain mucoadhesive properties, allowing attachment to the wet mucosal surface shortly after spraying.

Mechanistically, the hydrogel exhibits potent reactive oxygen species (ROS) scavenging capacity and intrinsic antibacterial activity, effectively remodeling the pathological wound microenvironment. Crucially, PDS Gel promotes macrophage polarization from a pro-inflammatory M1 phenotype toward a pro-regenerative M2 phenotype, thereby suppressing excessive inflammatory cascades. Simultaneously, it restores fibroblast-mediated extracellular matrix (ECM) remodeling and enhances endothelial cell-driven angiogenesis. In vivo evaluations using both oral mucosal and full-thickness skin defect models demonstrate that PDS Gel significantly accelerates wound closure, facilitates epithelial reconstruction, and promotes orderly collagen deposition. Collectively, this self-oxidatively crosslinked immunomodulatory hydrogel provides a promising biomaterial paradigm for the precision therapy of RAU and other complex mucosal lesions.

## Highlights

• Hierarchical crosslinking enables a sprayable and strongly adhesive hydrogel.
• The hydrogel regulates macrophage polarization and attenuates inflammation.
• Promotes fibroblast migration and endothelial angiogenesis.
• Enhances ECM remodeling and accelerates healing of recurrent ulcers.
• Accelerated healing is validated in both oral ulcer and skin wound models.

## Graphical abstract

**Figure:**
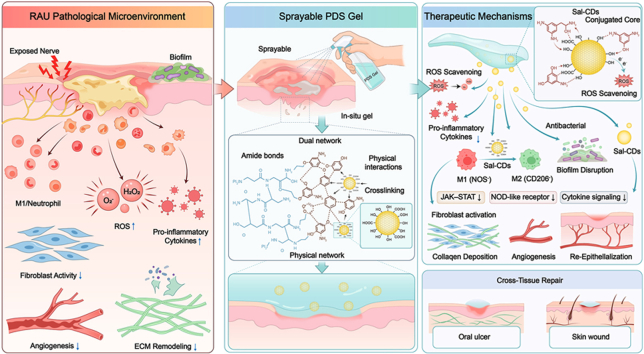

## 1 Introduction

Recurrent aphthous ulcer (RAU) is a common oral mucosal disease characterized by chronic recurrence and debilitating pain that severely impairs quality of life [1,2].

The persistent, hard-to-heal nature of RAU is driven by a severely dysregulated local microenvironment. Sustained inflammation, marked by immune cell infiltration and pro-inflammatory cytokines (e.g., TNF-α, IL-6), induces epithelial apoptosis and impairs repair [3]; excessive reactive oxygen species (ROS) exacerbate tissue damage through a bidirectional cycle with inflammation [4,5]; microbial colonization and biofilm formation further promote secondary infection [6]; while biofilm and microcirculatory disturbances limit nutrient and cellular supply necessary for regeneration [7]. The combined action of these factors ultimately suppresses fibroblast function and impairs extracellular matrix (ECM) remodeling, resulting in delayed healing, depressed (concave) repair, and increased recurrence risk [8]. Among these factors, the inflammatory–oxidative stress interplay is widely considered a key contributor to tissue injury and impaired repair. Therefore, developing therapeutic materials capable of multi-target regulation of this dysregulated microenvironment is of significant clinical importance.

Despite high clinical demand, conventional films and pastes fail due to rapid drug washout in the dynamic oral environment, leading to suboptimal bioavailability and frequent dosing [9]. Although hydrogels offer a promising biomimetic architecture for wound management, their clinical performance is often limited by several practical challenges [10]. Current smart hydrogel systems are frequently hindered by stringent crosslinking conditions. For instance, systems triggered by exogenous stimuli (e.g., UV light or non-physiological temperatures) are often incompatible with the fragile oral environment, potentially eliciting secondary mucosal irritation or localized tissue stress [[11], [12], [13]]. Furthermore, achieving immediate and firm wet adhesion on persistently hydrated mucosal surfaces remains challenging. Most existing materials serve merely as passive physical barriers, lacking the active, multi-dimensional metabolic reprogramming required to simultaneously neutralize the interrelated axes of ROS, sustained inflammation, and microbial infection.

To address the aforementioned challenges, this study aims to achieve breakthroughs at both the “material-design” and “functional-synergy” levels. Considering the highly dynamic and persistently hydrated environment of oral ulcers, polygalacturonic acid (PGA) and dopamine (DA) were selected as the hydrogel matrix. PGA, a natural polysaccharide, offers excellent biocompatibility, tunable degradability, and abundant carboxyl groups, enabling the construction of a compliant network that matches the mechanical properties of the oral mucosa [14,15]. DA, centered on catechol groups, not only mediates multimodal strong adhesion to the mucosal surface in wet conditions through hydrogen bonding, π–π interactions, metal coordination, and Schiff-base reactions [[16], [17], [18]], but its chemical structure also intrinsically confers antioxidant potential by donating hydrogen and facilitating electron transfer to scavenge ROS [19]. This provides an intrinsic, material-based therapeutic dimension for mitigating local ROS burden in ulcers, beyond conventional drug loading.

While the DA-modified matrix establishes a stable physical interface, the complex inflammatory–oxidative microenvironment of RAU necessitates additional bioactive intervention. Given its well-documented anti-inflammatory and antioxidant activity and rich phenolic structure, salidroside was selected as a representative natural therapeutic candidate for modulating the inflammatory–oxidative axis in RAU. Salidroside, a natural phenolic compound rich in oxygen-containing functional groups, possesses intrinsic antibacterial, anti-inflammatory, and antioxidant properties that align closely with RAU treatment requirements [20,21]. However, directly incorporating free salidroside into hydrogels often leads to rapid burst release and poor retention, limiting its effectiveness in dynamic oral environments [22,23]. Therefore, a structural transformation strategy rather than simple loading is required. To overcome this, we carbonized salidroside into carbon dots (Sal-CDs). Compared with the free compound, Sal-CDs retain partial bioactive functional groups and introduce a carbon-based structure with enhanced electron-transfer capability, which contributes to ROS scavenging [24]. Additionally, the abundant surface functional groups (e.g., –OH, –COOH) can further modulate pro-inflammatory signaling pathways, enabling coordinated action across both oxidative and inflammatory regulatory dimensions [25].

**Scheme 1:**
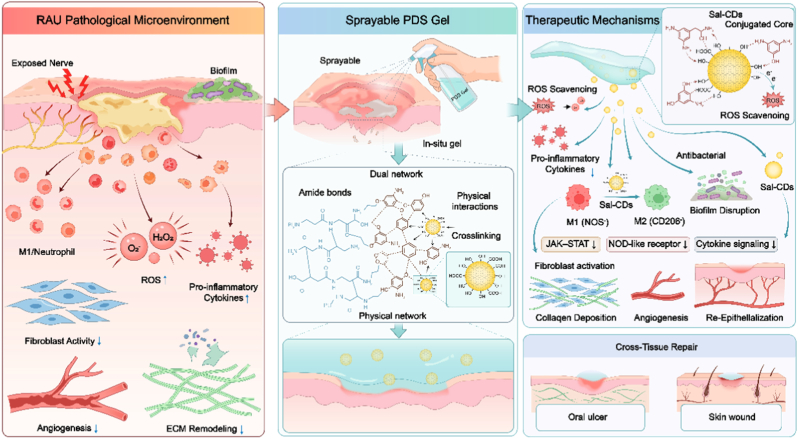
Schematic illustration of the pathological microenvironment of RAU and the therapeutic mechanism of sprayable PDS Gel.

Based on this, we developed a PDS smart hydrogel using a three-step strategy of “crosslinking innovation – functional integration – scenario expansion.” The resulting PGA-DA@Sal-CDs (PDS Gel) combines sprayability, self-crosslinking solidification, and multi-dimensional therapeutic functions. First, a hierarchical “oxygen-limited initiation – oxygen-controlled progression” crosslinking strategy was designed to balance flowability during spraying with in situ solidification on the wound, resolving the trade-off between operability and mechanical strength. Second, naturally derived Sal-CDs were stably integrated into the hydrogel via hydrogen bonding and coordination between their abundant –OH/–COOH groups and the amines and catechol groups of the dopamine oxidation network, achieving structural assembly and biological functionality (anti-inflammatory, antioxidant, antibacterial) in a single system. Finally, although oral mucosa and skin differ in ECM architecture, both are driven by similar inflammatory cascades and fibroblast-mediated ECM remodeling during wound healing [26]. Therefore, we evaluated the hydrogel in oral ulcer and skin defect models to preliminarily assess its translational potential for epithelial wound repair. The material maintains robust wet adhesion under simulated salivary flow and elicits effective early bioactivity, while promoting collagen remodeling and angiogenesis in skin wounds, demonstrating broad cross-tissue reparative potential and high translational value (Scheme 1).

In summary, this work establishes a multifunctional therapeutic platform that couples rapid in situ gelation with active microenvironment modulation. By addressing adhesion and multi-target regulation challenges in dynamic oral environments, this PDS system offers a promising strategy for treating complex epithelial wounds.

## 2 Materials and methods

### 2.1 Materials

Dopamine hydrochloride, N-hydroxysuccinimide (NHS), and sodium periodate were purchased from Aladdin (Shanghai, China).

Phosphate-buffered saline (PBS, pH 7.4), polygalacturonic acid, and 1-ethyl-3-(3-dimethylaminopropyl) carbodiimide hydrochloride (EDC) were purchased from Shanghai Macklin Biochemical Co., Ltd. (Shanghai, China).

2-Morpholino-ethanesulfonic acid hydrate (MES) was obtained from Sigma-Aldrich (St. Louis, USA).

Human umbilical vein endothelial cells (HUVECs), human gingival fibroblasts (HGFs), human dermal fibroblasts (HDFs), human oral keratinocytes (HOKs) and mouse macrophage-like RAW264.7 cells were obtained from the laboratory of the West China School of Stomatology, Sichuan University (Chengdu, China). *Escherichia coli* (*E. coli*) and *Staphylococcus aureus* (*S. aureus*) were purchased from Three Drugs Science and Technology Development Company (Beijing, China).

MES buffer (0.05 M, pH 6.0) was prepared by dissolving 9.762 g MES in 1 l of deionized water and the pH was adjusted to 6.

### 2.2 Preparation of Sal-CDs

Sal-CDs were synthesized via a one-step hydrothermal method. Briefly, 1.14 g of salidroside was dissolved in 40 mL of deionized water, corresponding to a precursor-to-solvent ratio of 1:35 (w/v), and transferred into a PTFE-lined stainless steel autoclave. The reaction was carried out at 180 °C for 10 h. After naturally cooling to room temperature, a brown-yellow solution was obtained. The solution was sonicated for 30 min and filtered through a 0.22 μm membrane to remove large particulates. The filtrate was then dialyzed in a 1000 Da MWCO dialysis bag against deionized water for 48 h, with water replaced every 6 h, to remove unreacted small molecules. Finally, the dialyzed solution was freeze-dried to yield brown-yellow Sal-CDs powder for subsequent use.

### 2.3 Preparation of PDS gel

PDS Gel was prepared via a stepwise crosslinking strategy. First, a PGA solution was activated by adding EDC and NHS at a molar ratio of PGA (carboxyl equivalent):EDC:NHS = 1:2:2. The pH was adjusted to ∼5.5 with MES buffer, and the reaction proceeded at room temperature for 10–15 min. After deoxygenation under inert gas, DA was added to a final concentration of 2 mg mL^−1^ to form the primary covalent crosslinked network.

For PDS Gel1, Sal-CDs were then added at 50 μg mL^−1^, and the mixture was allowed to stand for 10–15 min to generate a preliminary hydrogel network. Subsequently, the pH was adjusted to 7.4 with PBS, and the system was incubated under sealed conditions to promote further crosslinking via dopamine self-oxidation, yielding a pale yellow, semi-transparent hydrogel. PDS Gel2, PDS Gel3, and PDS Gel4 were prepared similarly, with Sal-CDs concentrations of 100, 150, and 200 μg mL^−1^, respectively.

### 2.4 Physicochemical characterization

The morphology of Sal-CDs was observed by transmission electron microscopy (TEM), and particle size distribution was analyzed using image analysis software. High-resolution TEM (HRTEM) was used to examine the lattice structure. The surface charge of Sal-CDs was measured using a Zeta potential analyzer to evaluate colloidal stability. X-ray diffraction (XRD, Cu Kα radiation, λ = 1.5406 Å, scanning range 2θ = 10°-100°) was performed to analyze the crystalline structure and reveal the graphitic characteristics of Sal-CDs. Fourier transform infrared spectroscopy (FT-IR, 4000–400 cm^−1^) was used to analyze the surface chemical structures of raw salidroside and Sal-CDs for comparison of their functional groups. The fluorescence quantum yield (QY) of Sal-CDs was measured using the relative method with quinine sulfate in 0.1 M H_2_SO_4_ as the reference (QY = 54%). The excitation wavelength was 360 nm. The integrated fluorescence intensity was plotted against the absorbance at 360 nm for both the reference and Sal-CDs, and the QY was calculated based on the slope ratio.

XRD was used to analyze the amorphous structure of the hydrogel network. FT-IR was employed to investigate chemical structural changes before and after hydrogel crosslinking. X-ray photoelectron spectroscopy (XPS) was conducted to analyze elemental composition and chemical states; high-resolution spectra of C 1s, N 1s, and O 1s were deconvoluted to elucidate bonding changes associated with hydrogel crosslinking and Sal-CDs incorporation. Based on a UV–vis absorbance–concentration calibration curve, the release kinetics of Sal-CDs from the hydrogel over 2 h were quantitatively determined. The microstructure of hydrogels was observed by scanning electron microscopy (SEM) after freeze-drying at −80 °C followed by gold sputtering. Rheological properties were evaluated using a rotational rheometer. Time sweep measurements recorded the evolution of storage modulus (G′) and loss modulus (G″) to determine the gelation point (defined as the time when G′ exceeded G″), while strain sweep tests (0.1%–100% strain) were conducted to assess network stability. Surface wettability was measured using a contact angle goniometer by placing a 5 μL droplet of deionized water on the hydrogel surface and recording the contact angle. Adhesion was evaluated using a finger model test: hydrogels were applied to the model finger surface, and adhesion stability was photographed at different orientations (0°, 90°, 180°, and 360°). Injectability was assessed by syringe extrusion at room temperature with observation of the extrusion morphology. For swelling analysis, the swelling behavior of PGA-DA and PDS Gel was first compared in PBS (pH 7.4). Hydrogel samples were immersed in PBS, retrieved at predetermined time points, gently blotted to remove surface water, and weighed. The swelling ratio was calculated as (W_t_ − W_0_)/W_0_, where W_0_ is the initial wet weight and W_t_ is the weight at each time point. To further simulate pH fluctuations in the oral environment, swelling and degradation experiments were conducted on PDS Gel using artificial saliva at pH 5.5, 6.5, and 7.5. The swelling test followed the same procedure as described above. For degradation, the hydrogels were incubated in artificial saliva, sampled at various time points, freeze-dried, and weighed to determine the mass retention rate. Fresh porcine oral mucosa was pre-wetted in artificial saliva at 37 °C for 15 min. Subsequently, PDS Gel was sprayed between two mucosal tissues (adhesion area: 15 mm × 10 mm), gently pressed for 30 s, and then left to stand for 2.5 min to allow gelation. Lap shear tests were then performed using a universal testing machine at a crosshead speed of 5 mm/min, and the stress–strain curve of lap shear was recorded and plotted.

### 2.5 Hydrogel biocompatibility evaluation

Hydrogel biocompatibility was systematically evaluated through in vitro cytocompatibility assays, hemocompatibility testing, and in vivo histopathological analysis.

HUVECs, HOKs, RAW264.7 cells, and fibroblasts (HDFs, HGFs, and L929) were seeded in 96-well plates (5 × 10^3^–2 × 10^4^ cells per well) and co-incubated for 24 h with blank PGA-DA hydrogels or PDS Gel containing different concentrations of Sal-CDs (PDS Gel1–4, 50, 100, 150, and 200 μg mL^−1^). Cell viability was determined using the CCK-8 assay, with the control group as baseline to evaluate proliferation promotion or inhibition. HUVECs and HOKs were further stained with Calcein-AM, and fluorescence images were captured at 0, 8, and 24 h to observe cell morphology and proliferation.

Fresh whole blood was incubated with the hydrogels, and absorbance was measured to calculate hemolysis rate. Blood compatibility was evaluated according to ISO 10993 standards (hemolysis rate <5%).

Major organs (heart, liver, spleen, lung, and kidney) from treated rats were collected and subjected to H&E staining to examine tissue structure and inflammatory responses, evaluating potential in vivo toxicity.

### 2.6 In vitro antibacterial evaluation

*E. coli* and *S. aureus* suspensions were diluted to ∼1 × 10^8^ CFU mL^−1^. Hydrogel samples (100 mg) were incubated with 1 mL bacterial suspension at 37 °C for 6 h. After serial dilution, samples were plated on LB agar and incubated for 12–16 h. Colony-forming units (CFUs) were counted to quantify antibacterial activity.

Bacteria treated with hydrogels for 24 h were stained using a SYTO9/PI kit according to the manufacturer's instructions. Samples were imaged with confocal laser scanning microscopy (CLSM), and fluorescence intensity was quantified to assess bacterial viability.

LB agar plates were evenly coated with bacteria. Sterile discs of uniform size were immersed in hydrogels for 10 s, placed on the plates, and incubated at 37 °C for 12–16 h. Inhibition zones were observed and photographed.

Pre-formed bacterial biofilms in 96-well plates were co-incubated with hydrogels for 6 h. Wells were washed to remove planktonic bacteria, stained with 0.1% crystal violet for 15 min, and dissolved in ethanol. Absorbance at 590 nm was measured to calculate biofilm disruption.

Treated bacteria were fixed in 2.5% glutaraldehyde for 2 h, dehydrated through a graded ethanol series (25%, 50%, 75%, 100%, 15 min each), dried, sputter-coated with gold, and observed by SEM to examine bacterial morphology.

For leakage assays, treated bacterial suspensions (*E. coli* and *S. aureus*, ∼1 × 10^8^ CFU mL^−1^) were incubated with different hydrogels at 37 °C for 6 h. Untreated bacterial suspensions served as the control. After incubation, samples were centrifuged (12,000 × g, 10 min, 4 °C), and the supernatants were collected for analysis. *β*-galactosidase leakage was measured using the ONPG (o-nitrophenyl-β-D-galactopyranoside) method by detecting absorbance at 420 nm (OD_420_). ATP, total protein, and DNA contents were quantified using a bioluminescence ATP assay kit, a BCA protein assay kit, and a PicoGreen dsDNA assay kit, respectively.

### 2.7 Evaluation of antioxidant and ROS-scavenging capacity

The overall antioxidant capacity of the materials was systematically evaluated using ABTS and DPPH radical scavenging assays.

ABTS was oxidized to generate blue ABTS**•^+^**, which shows a characteristic absorption peak at 734 nm. Antioxidant components in the hydrogel inhibit ABTS•^+^ formation, reducing absorbance. DPPH• in ethanol exhibits a purple color with strong absorbance at 517 nm. In the presence of antioxidants, DPPH• radicals are scavenged, leading to color fading and decreased absorbance. After incubation of PDS Gel with ABTS or DPPH solutions, ROS scavenging efficiency was calculated:

$$Scavenging\;rate\;\left(\%\right)=\frac{A_{control}-A_{sample}}{A_{control}}\times 100\%$$

The methylene blue (MB) assay was used to evaluate •OH clearance. MB shows a blue color with an absorption peak at 664 nm. •OH degrades MB, while antioxidants protect MB, maintaining higher absorbance. The scavenging rate was calculated as:

$$Scavenging\;rate\;\left(\%\right)=\frac{A_{sample}-A_{control}}{A_{\max}{-A}_{control}}\times 100\%$$

Horseradish peroxidase (HRP) + H_2_O_2_ + 3,3′,5,5′-tetramethylbenzidine (TMB) enzymatic system was used to assess catalase-mimetic activity of PDS Gel. In this system, HRP reacts with H_2_O_2_ to form high-activity intermediate Compound I, which oxidizes colorless TMB to a blue product with an absorption peak at 652 nm. Antioxidant materials inhibit this H_2_O_2_-mediated oxidation, resulting in decreased color and absorbance. The inhibition rate was calculated as:

$$Inhibition\;Rate\;\left(\%\right)=\frac{A_{control}-A_{sample}}{A_{control}}\times 100\%$$

To evaluate intracellular delivery and distribution, HUVECs were co-cultured with Sal-CDs at 50, 100, 150, and 200 μg mL^−1^ for 24 h. After washing with PBS, confocal microscopy was used to observe intracellular distribution, and fluorescence intensity was quantified using ImageJ to assess uptake efficiency.

The ROS-scavenging ability of PDS Gel in cells was investigated using the DCFH-DA probe. DCFH-DA is a cell-permeable, non-fluorescent probe that is hydrolyzed intracellularly to DCFH, which is trapped in cells. DCFH is oxidized by ROS to fluorescent DCF. HUVECs, RAW264.7 cells, and HGFs were seeded in 24-well plates (2 × 10^4^ cells/well) and ROS production was induced with LPS (for macrophages) or H_2_O_2_ (for HUVECs and HGFs). Cells were then treated with PDS Gel containing different concentrations of Sal-CDs for 24 h. After washing with PBS, cells were incubated with DCFH-DA for 30 min, washed three times with basal medium, and ROS fluorescence intensity was observed using confocal microscopy.

### 2.8 In vitro cell studies

#### 2.8.1 Macrophage polarization assay

RAW264.7 cells were used to evaluate the modulatory effects of hydrogels on macrophage polarization. Cells were seeded at 2 × 10^4^ cells/well in 24-well plates. After attachment, groups were set as: Blank (unstimulated cells without any treatment), Control (cells stimulated with 1 μg mL^−1^ LPS to induce an inflammatory model, no hydrogel treatment), and treatment groups (cells stimulated with 1 μg mL^−1^ LPS and concurrently treated with PGA-DA, PDS Gel2, or PDS Gel3) for 24 h.

Immunofluorescence (IF) staining: Cells were fixed, permeabilized, and blocked, then incubated with primary antibodies against iNOS and CD86 (M1 markers) or CD206 (M2 marker), followed by fluorescent secondary antibodies. Images were captured using CLSM, and fluorescence intensity was quantified using ImageJ.

Enzyme-linked immunosorbent assay (ELISA): Protein levels of iNOS, CD206 in cell supernatants were quantified using commercial ELISA kits according to the manufacturer's instructions. Absorbance was measured with a microplate reader, and concentrations were calculated based on standard curves.

Quantitative real-time PCR (qRT-PCR): Total RNA was extracted and reverse-transcribed into cDNA. qRT-PCR was performed to measure M1-associated genes (iNOS, TNF-α, IL-6, IL-1β) and M2-associated genes (Arg-1, CD206, IL-10), using GAPDH or β-actin as internal references. Relative expression was calculated by the 2^-ΔΔCt^ method. Primer sequences are listed in Table S1.

Transcriptome sequencing: RAW264.7 cells stimulated with LPS and treated with PDS Gel3 were subjected to RNA-seq. Differentially expressed genes were identified and visualized using volcano plots and heatmaps. Gene Ontology (GO) enrichment and Kyoto Encyclopedia of Genes and Genomes (KEGG) pathway analyses were performed. Gene set enrichment analysis (GSEA) was applied to evaluate key pathways including JAK-STAT signaling, NOD-like receptor signaling, cytokine–cytokine receptor interaction, and oxidative phosphorylation.

#### 2.8.2 Angiogenesis-related assays

HUVECs were used to systematically evaluate the effects of PDS Gel on angiogenesis-related behaviors. Cells were seeded in 24-well plates (2 × 10^4^ cells/well) and divided into: Blank (unstimulated), Control (LPS-induced inflammation), PGA-DA (LPS + PGA-DA hydrogel), PDS Gel2 (LPS + PDS Gel2), and PDS Gel3 (LPS + PDS Gel3) groups for 24 h.

Transwell migration assay: After 24 h treatment, cells were collected and seeded into the upper chamber of Transwell inserts, with complete medium in the lower chamber. Following 12–16 h incubation, migrated cells were fixed, stained with crystal violet, and counted in three randomly selected fields under a microscope.

Scratch wound assay: HUVECs were cultured to confluence in 6-well plates. Linear scratches were made with a 200 μL pipette tip, followed by treatment with respective hydrogels. Images were captured at 0, 8, and 12 h, and wound width and closure rate were quantified using ImageJ.

Tube formation assay: 96-well plates were pre-coated with Matrigel (50 μL/well) and polymerized at 37 °C for 30 min. Treated HUVECs (2 × 10^4^ cells/well) were seeded onto Matrigel and incubated for 6–8 h. Five random fields per well were imaged, and tube number, branch points, and total tube length were quantified using ImageJ.

VEGF expression analysis: After 24 h treatment, cells were fixed, permeabilized, and blocked, then incubated with *anti*-VEGF primary antibody and fluorescent secondary antibody. Nuclei were counterstained with DAPI. Confocal images were acquired, and VEGF fluorescence intensity was quantified using ImageJ.

The secretion levels of VEGF in the culture supernatant were quantitatively analyzed using commercial ELISA kits according to the manufacturer's instructions. Absorbance was measured with a microplate reader, and the concentrations were determined by interpolation from standard curves.

#### 2.8.3 Fibroblast-related assays

To assess the regulatory effects of hydrogels on fibroblast migration and tissue-repair functions under inflammatory conditions, HGFs and HDFs were seeded in 6-well plates and cultured to confluence. Inflammation was induced by treating cells with LPS (1 μg mL^−1^) for 24 h. Experimental groups included Control (LPS), PGA-DA (LPS + PGA-DA), and PDS Gel (LPS + PDS Gel).

Scratch wound assay: Linear scratches were made on the cell monolayer with a sterile pipette tip. Floating cells were removed, and low-serum medium with respective treatments was added. Images were captured at 0, 24, and 48 h, and wound area and closure rate were quantified using ImageJ.

IF staining: Treated cells were fixed, permeabilized, and blocked, then incubated with primary antibodies against α-SMA, COL1A1, and FN1, followed by fluorescent secondary antibodies. Confocal images were acquired, and fluorescence intensity was quantified.

Gene expression analysis: Total RNA from HDFs was extracted and reverse-transcribed into cDNA. qRT-PCR was performed to measure mRNA levels of α-SMA, TGF-β1, COL1A1, FN1, and VEGF, using GAPDH as the internal reference. Relative expression was calculated using the 2^-ΔΔCt^method.

### 2.9 Animal experiments

Sprague–Dawley (SD) rats were obtained from the Experimental Animal Center of Shanxi Medical University. All animal experiments were approved by the Animal Ethics Committee of Shanxi Medical University (Ethics Approval No.: KQDW-2024-004). Procedures were strictly conducted in accordance with the UK Animals (Scientific Procedures) Act 1986 and its relevant guidelines, and complied with the ethical review requirements of the institutional committee.

#### 2.9.1 Rat oral ulcer model

To evaluate the adhesion duration under dynamic oral conditions, an in vitro dynamic flushing model was established. The PDS Gel precursor solution was sprayed onto the surface of two pieces of ex vivo porcine oral mucosa, which were then pressed together for 5 min to allow gel crosslinking. The sandwiched hydrogel was subsequently immersed in artificial saliva at different pH values (5.5, 6.5, and 7.4) and maintained at 37 °C under orbital shaking at 60 rpm. The time until detachment of the two mucosal pieces was recorded as the adhesion failure time.

To further verify the adhesion performance in a more realistic oral environment, FITC-labeled PDS Gel was sprayed onto the surface of rat tongues. Fluorescence observation was performed at different time points to monitor the retention of the hydrogel.

RAU model and treatment: SD rats were anesthetized with sodium pentobarbital. A circular filter paper (3 mm diameter) soaked in 50% (v/v) acetic acid was applied to the left buccal mucosa for 40 s, followed by saline rinse. Primary ulcers formed within 24 h and healed completely by day 7. After a 7-day recovery, the procedure was repeated at the same site to induce recurrent ulcers, simulating the clinical phenotype. Treatment was administered according to the experimental groups three times daily (100 μL per dose) with a 6-h interval between doses, and ulcer images were recorded on days 0, 2, 4, and 6. On day 6, rats were sacrificed, and ulcer tissues were collected for H&E, Masson staining, and IF/immunohistochemistry (IHC) analysis.

#### 2.9.2 Full-thickness skin wound model in rats

Male SD rats were anesthetized via intraperitoneal injection of 1% pentobarbital sodium after dorsal hair removal and iodine disinfection. Full-thickness skin defects (8 mm diameter) were created symmetrically on both sides of the dorsal midline using a sterile skin punch, excising skin down to the underlying muscle fascia. Immediately after wound creation, 50 μL of *S. aureus* suspension (1 × 10^8^ CFU mL^-1^) was applied and left for 30 min to promote bacterial colonization. Treatments were then initiated according to grouping. During the early inflammatory phase (days 0–5 post-op), the hydrogel (100 μL) was applied twice daily with a 6-h interval to cover the wound and margins. For the Sal-CDs group, an equivalent dose of Sal-CDs in saline was sprayed onto the wound. Once a stable scab formed, the material was applied to the wound margins and adjacent areas once daily until healing. Wound images were recorded on days 0, 4, 7, 10, and 14, and wound areas were measured using ImageJ. On day 7, wound tissues were collected for *S. aureus* colony counting. On day 14, animals were sacrificed, and wound tissues were harvested for H&E, Masson trichrome, Sirius Red staining, and IF/IHC analyses.

### 2.10 Statistical analysis

All quantitative data are presented as mean ± standard deviation (SD). Statistical analyses were performed using GraphPad Prism (version 9). For comparisons among multiple groups, one-way analysis of variance (ANOVA) followed by Tukey's post hoc test was applied.

All in vitro experiments were independently repeated at least three times (n = 3), and in vivo experiments were conducted using six animals per group (n = 6).

Statistical significance was defined as ns (*P* > 0.05), ∗*P* < 0.05, ∗∗*P* < 0.01, ∗∗∗*P* < 0.001, and ∗∗∗∗*P* < 0.0001.

## 3 Results and discussion

### 3.1 Successful fabrication of Sal-CDs and hydrogel

**Fig. 1:**
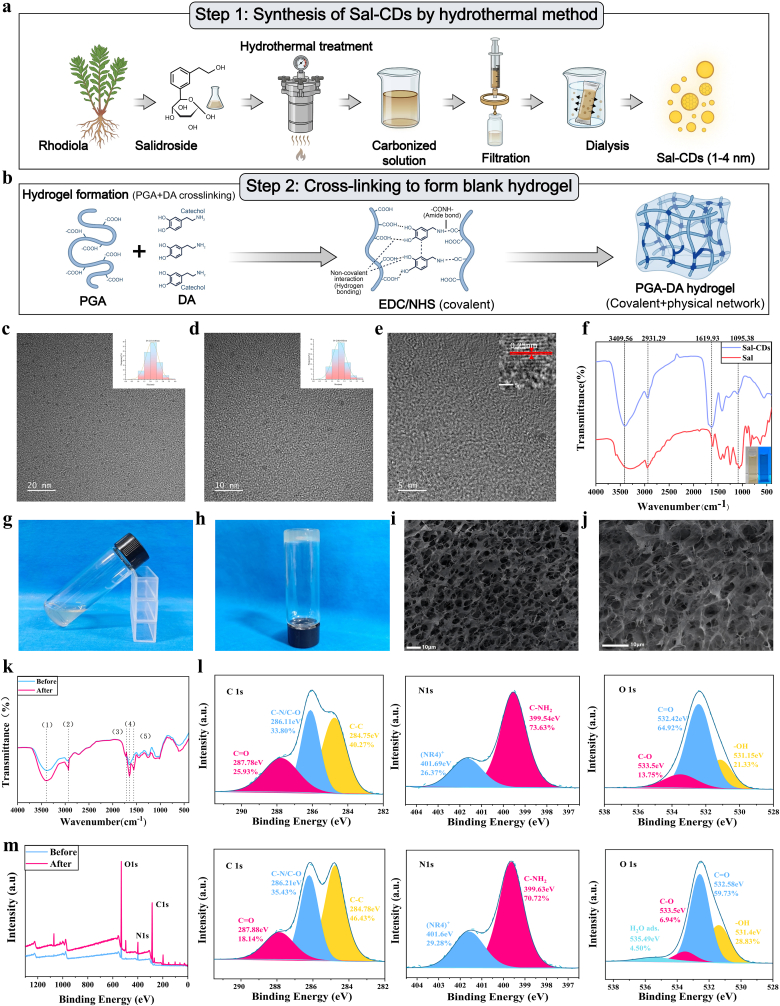
**Preparation and characterization of Sal-CDs and PGA-DA.** (a) Schematic illustration of the preparation process of Sal-CDs. (b) Schematic illustration of the preparation process of PGA-DA. (c–d) TEM images of Sal-CDs. (e) HRTEM image of Sal-CDs. (f) FTIR spectra of salidroside (Sal) and Sal-CDs, along with their fluorescence images under natural light and UV light. (g, h) Photographs of PGA-DA before and after gelation. (i, j) SEM images of PGA-DA. (k) FTIR spectra of PGA-DA before and after crosslinking. The characteristic peaks correspond to (1) O–H/N–H stretching, (2) C–H stretching, (3) carboxyl C=O stretching, (4) amide I band, and (5) amide II band. (l, m) XPS spectra of PGA-DA before and after crosslinking.

Sal-CDs were synthesized via a one-step hydrothermal method using salidroside as the carbon source (Fig. 1a). TEM images showed that the Sal-CDs were uniform and spherical, with diameters mainly ranging from 1 to 4 nm and an average size of 2.13 ± 0.01 nm (Fig. 1c and d), exhibiting good dispersibility. HRTEM images revealed clear lattice fringes with a spacing of approximately 0.25 nm (Fig. 1e), indicating partial graphitic sp^2^ carbon domains [27]. The Zeta potential of Sal-CDs was −14.49 mV (Fig. S1), exhibiting weak negative charge due to deprotonation of surface oxygen-containing groups in aqueous environments, which generally favors stability and biocompatibility. XRD patterns (Fig. S2) displayed a broad peak at 2θ ≈ 27.6°, corresponding to an interlayer spacing of ∼3.23 Å, attributable to (002) plane stacking of graphitic carbon (∼3.35 Å, JCPDS No. 75-1621), indicating partial short-range ordered graphitic structure [28]. The pronounced peak broadening and elevated background suggested that Sal-CDs are predominantly amorphous. Thus, Sal-CDs can be regarded as nanocarbon materials composed of partially graphitized domains embedded in an amorphous carbon matrix, consistent with typical carbon dot XRD features [29]. Fluorescence characterization revealed that the fluorescence quantum yield was measured to be 27.8% using the relative reference method (Fig. S3). To further verify the successful carbonization of salidroside into Sal-CDs, the FTIR spectra of raw salidroside and Sal-CDs were compared (Fig. 1f). Raw salidroside exhibited a broad and intense absorption peak at 3409 cm^−1^, attributed to O–H stretching vibrations arising from the dense hydrogen-bonding network of its glycosidic structure. After carbonization, this peak became markedly narrower, indicating that the hydrothermal process disrupted the original hydrogen-bonding network, while residual hydroxyl groups remained in a more free state [30]; Raw salidroside also showed strong aliphatic C–H stretching vibrations at 2931 cm^−1^ and intense glycosidic C–O–C/C–OH stretching bands in the 1000–1100 cm^−1^ region. These characteristic peaks were weakened in Sal-CDs, suggesting that the glycosidic backbone and aliphatic chains underwent cleavage and dehydration during carbonization [31]. Meanwhile, a prominent absorption band appeared at 1619 cm^−1^ in Sal-CDs, originating from aromatic C=C skeletal vibrations and conjugated C=O/–COO^-^ structures, whereas raw salidroside showed relatively weaker absorption in this region. This confirms the formation of graphitic-like structures with conjugated sp^2^ carbon domains after carbonization [32,33], indicating that salidroside was carbonized and introduced carboxyl functional groups during the hydrothermal process. In addition, Sal-CDs retained certain absorption features in the 1000–1100 cm^−1^ region, suggesting that some glycosidic oxygen-containing structures of the precursor were inherited and rearranged on the carbon dot surface [34]. Collectively, these comparative results directly demonstrate that salidroside was successfully converted into Sal-CDs via hydrothermal carbonization, and that the resulting carbon dots possess abundant hydroxyl, carboxyl, and residual glycoside-related surface groups, providing a structural basis for subsequent crosslinking with PGA-DA hydrogel and related biological functions.

The PGA-DA hydrogel was successfully constructed via EDC/NHS-mediated amidation, forming a porous three-dimensional network stabilized by both covalent crosslinks and non-covalent interactions (Fig. 1b). The crosslinked system (Fig. 1h) exhibited a stable, non-flowing gel state, in sharp contrast to the pre-crosslinked solution, which remained fluid (Fig. 1g). SEM images revealed a uniform and interconnected porous microstructure resulting from this crosslinking (Fig. 1i and j).

The chemical evolution of the hydrogel was further analyzed by FT-IR and XPS. FT-IR spectra (Fig. 1k) showed enhanced amide I band at 1650.78 cm^−1^ and a new characteristic amide II peak at 1558.21 cm^−1^, directly confirming the formation of amide bonds between PGA carboxyl groups and dopamine amino groups. The broadening of the O–H/N–H absorption band (3200–3500 cm^−1^) further indicated strengthened intermolecular hydrogen bonding within the network.

XPS analysis corroborated the FT-IR findings (Fig. 1l and m). The N 1s spectrum showed a decrease in –NH_2_ components, confirming partial participation of amino groups in amide bond formation; the C 1s spectrum exhibited an increased C–C/C–H component, reflecting the incorporation of dopamine's aromatic structure; and the O 1s spectrum showed an increase in –OH-related components, indicating a higher relative proportion of oxygen-containing groups capable of participating in non-covalent interactions after crosslinking.

**Fig. 2:**
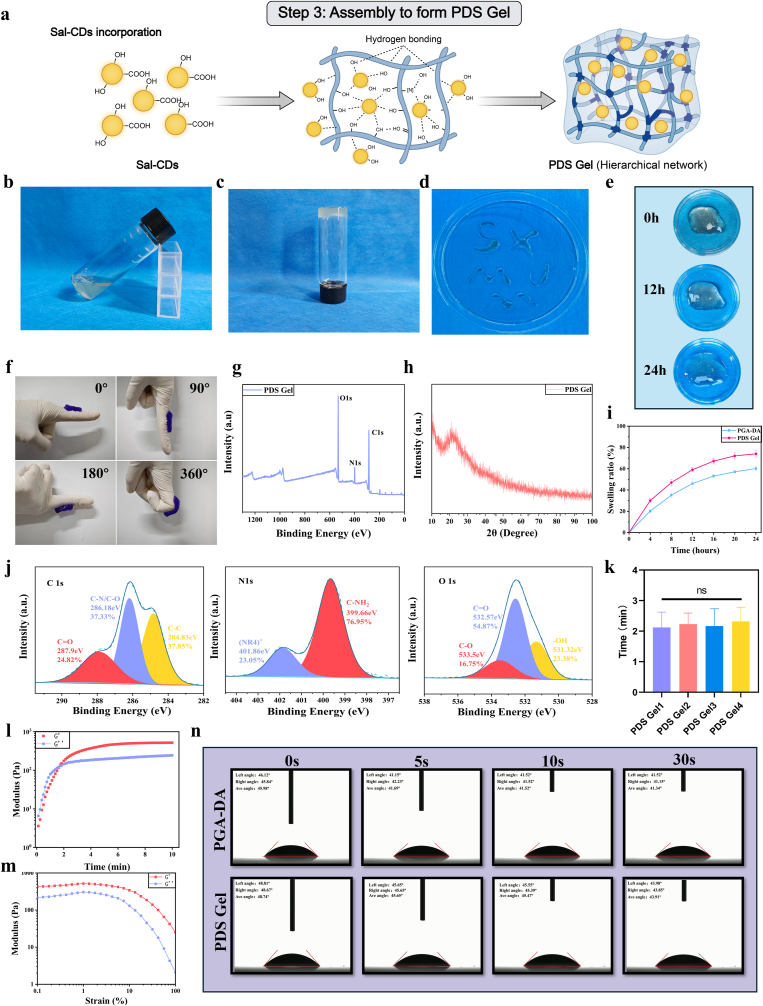
**Synthesis and characterization of PDS Gel.** (a) Assembly of Sal-CDs and PGA-DA to form PDS Gel. (b, c) Photographs of PDS Gel before and after gelation. (d–f) Digital photographs showing the injectability (d), swelling (e), and adhesiveness (f) of PDS Gel at room temperature. (g) XPS survey spectrum of PDS Gel. (h) XRD pattern of PDS Gel. (i) Swelling curves of PGA-DA and PDS Gel. (j) XPS high-resolution spectra of PDS Gel. (k) Gelation time of PDS Gel with different Sal-CDs contents. (l) Rheological time sweep showing the gelation process of PDS Gel. (m) Strain sweep test evaluating the mechanical stability and network integrity of the hydrogel (37 °C). (n) Water contact angle images of PGA-DA and PDS Gel. Data are presented as mean ± SD (n = 3). Statistical analysis was performed using one-way ANOVA followed by Tukey's post hoc test. ∗*P* < 0.05, ∗∗*P* < 0.01, ∗∗∗*P* < 0.001, and ∗∗∗∗*P* < 0.0001.

Following the introduction of Sal-CDs, the assembly of the PDS Gel hydrogel was completed (Fig. 2a), and its macroscopic crosslinked state remained unaffected (Fig. 2b and c). XPS analysis (Fig. 2g and j) indicated that, compared with PGA-DA hydrogel, the proportion of C–N/C–O increased from 33.80% to 37.33%, while the O 1s spectrum showed a significant increase in –OH and C–O components. This suggests that Sal-CDs were incorporated into the hydrogel network mainly through hydrogen bonding and coordination interactions between their abundant surface hydroxyl and carboxyl groups and the PGA/DA chains, which is consistent with their limited release behavior within 2 h (Fig. S4). XRD analysis revealed that PDS Gel exhibited a broad peak pattern similar to that of PGA-DA hydrogel (Fig. 2h), indicating that the overall hydrogel structure remained predominantly amorphous. Importantly, no diffraction peaks attributable to Sal-CDs or other crystalline phases were detected, demonstrating that Sal-CDs were highly dispersed in an amorphous state within the network without introducing new crystalline phases or undergoing phase separation. These findings further confirm the excellent structural compatibility between Sal-CDs and the PGA-DA hydrogel network.

For effective application in oral ulcers, hydrogels require strong adhesiveness. As shown in Fig. 2f, PDS Gel adhered well to a model finger and could be rotated without detachment, demonstrating excellent adhesive performance. Lap shear tests performed on porcine oral mucosa showed that the PDS hydrogel reached a peak shear strength of approximately 10.59 kPa. The corresponding stress–strain curve exhibited a broadened peak region at around 75–80% strain, followed by a gradual decrease in stress (Fig. S5). This curve profile suggests that the hydrogel was able to sustain stable interfacial contact with the wet mucosal surface over a considerable deformation range under shear loading, reflecting reliable wet adhesion performance. The hydrogel also exhibited good injectability at room temperature (Fig. 2d). Swelling behavior was further investigated. Compared with PGA-DA hydrogel, PDS Gel exhibited slightly higher swelling ratios at all time points, which can be attributed to the introduction of Sal-CDs that increased the content of hydrophilic functional groups, thereby enhancing water molecule adsorption. Notably, PDS Gel still maintained a moderate to low swelling level, indicating that the stepwise crosslinked network was not compromised by the incorporation of functional components (Fig. 2e and i). Considering the fluctuation of oral pH under inflammatory conditions (pH 5.5–7.5), we further evaluated the swelling behavior of PDS Gel within this pH range (Fig. S6). The swelling ratio varied slightly across different pH conditions but remained at low to moderate levels, with no excessive swelling or structural disintegration. Consistent with the in vitro degradation results (Fig. S6), PDS Gel exhibited slow and controllable degradation at all tested pH values while retaining an intact gel morphology. These findings demonstrate that PDS Gel maintains good dimensional and structural stability across the physiological and pathological pH range of the oral cavity, thereby supporting its stable structure during the critical early treatment window after application.

As a self-oxidatively crosslinked hydrogel, the crosslinking time of PDS Gel is a critical parameter. Rheological measurements revealed a typical time-dependent evolution of the storage modulus (G′) and loss modulus (G″). At the initial stage, G″ was slightly higher than G′, indicating a viscous-dominated fluid state that allowed sprayability. As the crosslinking reaction proceeded, G′ gradually increased and eventually exceeded G″, indicating the formation of a hydrogel network and the transition to an elasticity-dominated solid state during oxidative crosslinking. The time corresponding to this transition is defined as the gel point, reflecting the key kinetics of network formation (Fig. 2l). Statistical analysis further showed that the incorporation amount of Sal-CDs had little effect on the crosslinking time of PDS Gel, which remained within 3 min, meeting the requirements for clinical application (Fig. 2k).

Strain sweep tests were performed to evaluate the structural stability and deformation resistance of the hydrogel under different strain conditions (Fig. 2m). Within the strain range of 0.1%–100%, the storage modulus (G′) consistently remained higher than the loss modulus (G″), indicating that the material maintained a predominantly elastic gel state throughout the test. With further increases in strain, both G′ and G″ gradually decreased, yet no abrupt modulus collapse or G′/G″ crossover occurred, suggesting that the hydrogel did not undergo instantaneous structural failure under large deformation but instead maintained network continuity through gradual dissipation of strain energy. This progressive mechanical response indicates that the hydrogel can effectively accommodate external mechanical disturbances and maintain structural stability during repeated deformation or application, which is beneficial for stable adhesion and functional stability under repeated short-term mechanical disturbance such as oral mucosa or skin wounds.

To further investigate the hydrophilicity of PDS Gel, its water contact angle was measured (Fig. 2n). Both PGA-DA and PDS Gel exhibited relatively low water contact angles (<50°), indicating good surface hydrophilicity. Over time, water droplets gradually spread across the hydrogel surface, accompanied by a continuous decrease in contact angle, reflecting favorable wettability. Notably, PDS Gel consistently exhibited lower contact angles throughout the measurement, which may be attributed to the introduction of Sal-CDs-related functional groups that increase surface polarity, thereby further enhancing hydrophilicity. This property facilitates rapid spreading and stable adhesion of the hydrogel in moist wound environments.

Collectively, PDS Gel forms a hierarchical network structure through amide-based covalent crosslinking combined with dopamine–carbon dot synergistic physical crosslinking, providing a molecular foundation for its structural stability and functional performance.

### 3.2 Biocompatibility of the hydrogel

Given that PDS Gel is intended to directly contact oral mucosa or wound tissues during application, its biosafety is a fundamental prerequisite for practical use. Based on the structural and physicochemical characterizations described above, its biocompatibility was further evaluated at both the in vitro cellular level and the in vivo tissue level to assess its potential effects on relevant cell viability and major organ tissues.

CCK-8 assays showed that within the tested concentration range, PGA-DA and PDS Gel1–3 loaded with low to moderate amounts of Sal-CDs (50, 100, and 150 μg mL^−1^) exhibited no cytotoxicity toward mouse fibroblasts (L929), HDFs, HGFs, and macrophages (RAW264.7 cells). Cell viability remained higher than that of the control group, indicating a certain proliferation-promoting effect. In contrast, the high-loading formulation PDS Gel4 slightly inhibited the proliferation of these cells, suggesting a possible concentration-dependent biological effect of Sal-CDs (Fig. S7c–f).

In HUVECs and HOK, all material groups generally promoted cell proliferation, among which PDS Gel3 exhibited the most pronounced effect, while PDS Gel4 showed relatively weaker proliferative activity but no obvious cytotoxicity (Fig. S7a and b). Live/dead staining using AM/Calcein further confirmed that cells in all treatment groups maintained intact morphology and high viability at 0, 8, and 24 h, consistent with the CCK-8 results (Fig. S7g).

Hemolysis tests indicated that all material groups exhibited hemolysis rates below 5%, meeting the hemocompatibility standards specified by ISO 10993 (Fig. S7h). In vivo biosafety assessment showed that H&E staining of the heart, liver, spleen, lung, and kidney tissues from treated rats revealed no evident inflammation or tissue damage, with no significant differences compared to the control group (Fig. S8).

Taken together, the in vitro cellular assays, hemolysis tests, and in vivo histopathological analyses indicate that PGA-DA hydrogel and PDS Gel1–3 loaded with appropriate concentrations of Sal-CDs exhibit good biocompatibility and cytocompatibility. In contrast, excessive Sal-CDs loading may reduce the proliferation-promoting effects on certain cell types. Therefore, PDS Gel1–3 were selected for subsequent investigations of biological properties.

### 3.3 Antibacterial performance of the hydrogel

Previous studies have shown that ulcerative wounds are highly susceptible to rapid colonization by resident oral microbiota, which can subsequently form structurally stable bacterial biofilms, leading to secondary infections, prolonged inflammation, and delayed tissue repair [35]. Therefore, the ability of a material to effectively inhibit bacterial growth and disrupt biofilm formation, while maintaining biocompatibility, is a key criterion for evaluating its practical value as a treatment for oral ulcers. Based on this, the present study systematically assessed the antibacterial performance of PGA-DA hydrogel and PDS Gel1–3 loaded with varying concentrations of Sal-CDs against the Gram-negative bacterium *E. coli* and the Gram-positive bacterium *S. aureus*.

**Fig. 3:**
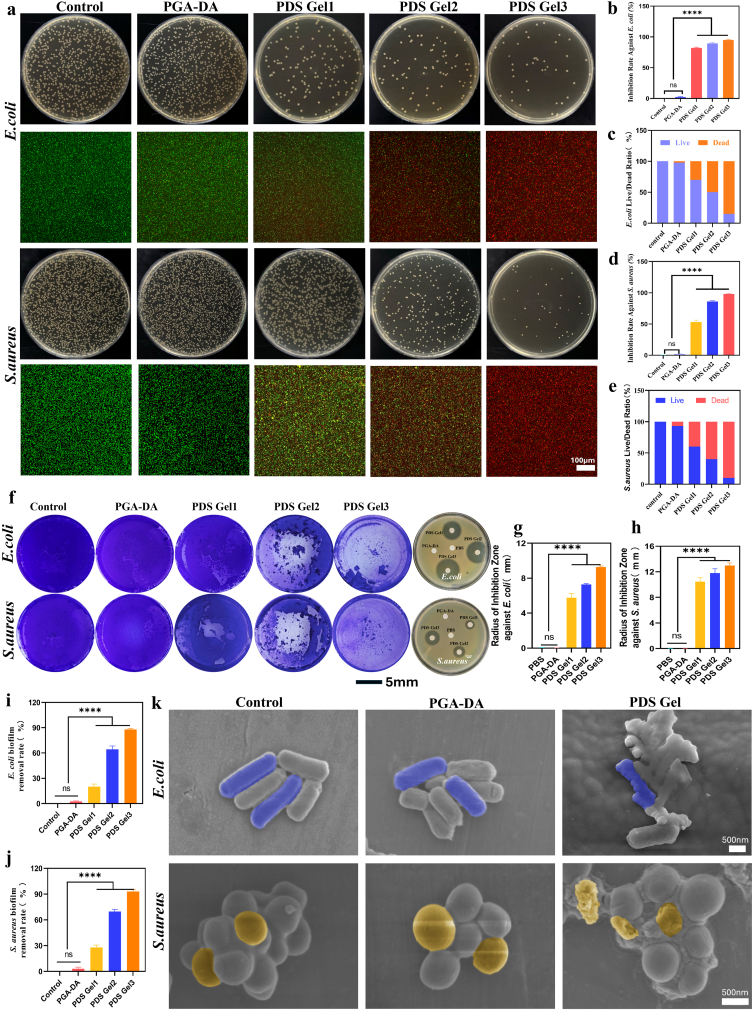
**Antibacterial performance of PDS Gel.** (a) Antibacterial activity of PDS Gel evaluated by plate colony counting and live/dead bacterial staining. (b, c) Colony counting of *E. coli* and quantitative analysis of bacterial viability based on live/dead fluorescence staining corresponding to (a). (d, e) Colony counting of *S. aureus* and quantitative analysis of bacterial viability based on live/dead fluorescence staining corresponding to (a). (f) Representative images of bacterial biofilms and inhibition zones under different treatments. (g, h) Quantitative analysis of inhibition zone radius corresponding to (f). (i, j) Quantitative analysis of biofilm disruption efficiency corresponding to (f). (k) SEM images of bacteria under different treatments. Data are presented as mean ± SD (n = 3). Statistical analysis was performed using one-way ANOVA followed by Tukey's post hoc test. ∗*P* < 0.05, ∗∗*P* < 0.01, ∗∗∗*P* < 0.001, and ∗∗∗∗*P* < 0.0001.

Colony counting results (Fig. 3a,b,d) showed that PGA-DA exhibited minimal inhibitory effect against both bacteria, whereas PDS Gel demonstrated progressively enhanced antibacterial activity with increasing Sal-CDs loading, with PDS Gel3 showing the most pronounced inhibition of *E. coli* and *S. aureus*. Live/dead bacterial staining results were consistent with these trends (Fig. 3a,c,e): the PGA-DA group predominantly displayed green live bacteria, while PDS Gel treatment markedly increased the red dead bacteria signal, which intensified with higher Sal-CDs concentrations. These findings indicate that the incorporation of Sal-CDs effectively endows the hydrogel with robust antibacterial activity.

Further biofilm experiments revealed that PGA-DA had minimal effect on pre-formed bacterial biofilms, whereas PDS Gel1–3 markedly disrupted the structural integrity of *E. coli* and *S. aureus* biofilms, with the effect intensifying as Sal-CDs content increased. Among them, PDS Gel3 exhibited the most pronounced biofilm disruption (Fig. 3f,i,j). Scanning electron microscopy further illustrated morphological changes of bacteria following different treatments (Fig. 3k): bacteria in the PGA-DA group maintained intact morphology with smooth surfaces, while PDS Gel-treated bacteria showed evident membrane collapse, surface wrinkling, and structural rupture. Consistent with these observations, the leakage of β-galactosidase, ATP, protein, and DNA was quantified (Fig. S9), confirming concentration-dependent membrane disruption by PDS Gel. Consistently, inhibition zone assays confirmed these antibacterial trends, with no clear inhibition zone around PGA-DA, whereas PDS Gel1–3 generated distinct and progressively enlarging inhibition zones (Fig. 3f–h).

In the antibacterial evaluation against *E. coli* and *S. aureus*, PDS Gel loaded with Sal-CDs exhibited evident inhibitory effects, accompanied by pronounced abnormalities in bacterial membrane morphology. Scanning electron microscopy revealed that treated bacteria displayed membrane collapse, wrinkling, and structural disruption, indicating severe impairment of membrane function. Notably, Sal-CDs exhibited only a weak negative zeta potential (−14.49 mV), suggesting that their antibacterial activity does not rely on strong cationic surface-induced electrostatic adsorption or direct membrane rupture. Considering the material's origin and recent studies on the antibacterial mechanisms of carbon dots, this effect is likely driven by the synergistic contribution of multiple non-electrostatic interactions [36]. Previous research has shown that *Rhodiola rosea* and its primary active component salidroside possess inherent antibacterial potential, capable of inhibiting bacterial growth by interfering with metabolic processes and membrane-associated functions [37]. In this study, salidroside served as the carbon source for the carbon dots; although its molecular structure undergoes reorganization during carbonization, some aromatic structures and oxygen-containing functional groups are retained, providing a baseline of bioactivity for Sal-CDs. Furthermore, recent studies indicate that the antibacterial activity of carbon dot-based nanomaterials generally arises from multiple non-electrostatic mechanisms, including interactions with bacterial membranes and intracellular components, as well as modulation of metabolic processes and redox homeostasis, rather than solely relying on surface charge [[38], [39], [40]]. Under conditions of weakly negative surface potential, these combined effects can still compromise bacterial membrane stability, and the membrane collapse and rupture observed via SEM likely represent terminal morphological phenotypes following bacterial dysfunction and death.

In summary, PGA-DA itself exhibits negligible antibacterial activity, whereas the incorporation of Sal-CDs endows the hydrogel with concentration-dependent antibacterial effects against *E. coli* and *S. aureus* as well as biofilm-disrupting capabilities. By effectively inhibiting bacterial colonization and infection spread while maintaining excellent biocompatibility, PDS Gel provides critical functional support for preventing secondary infections, improving the local microenvironment, and promoting tissue repair in RAU lesions.

### 3.4 Antioxidant and ROS-scavenging capacity of the hydrogel

Having demonstrated the antibacterial activity of PDS Gel, we next examined its antioxidant capacity. The persistent nature of RAU is closely associated with local oxidative stress imbalance. Excessive ROS accumulation induced by inflammatory stimuli can not only directly damage epithelial and stromal cells but also amplify inflammatory signaling pathways, thereby inhibiting fibroblast migration and tissue regeneration. Therefore, the ability of a material to modulate the oxidative microenvironment and mitigate inflammation-associated ROS burden, while maintaining biocompatibility, represents a critical dimension for evaluating its therapeutic potential [41]. Based on this, the present study systematically assessed the antioxidant performance of PDS Gel at both in vitro chemical and cellular levels.

**Fig. 4:**
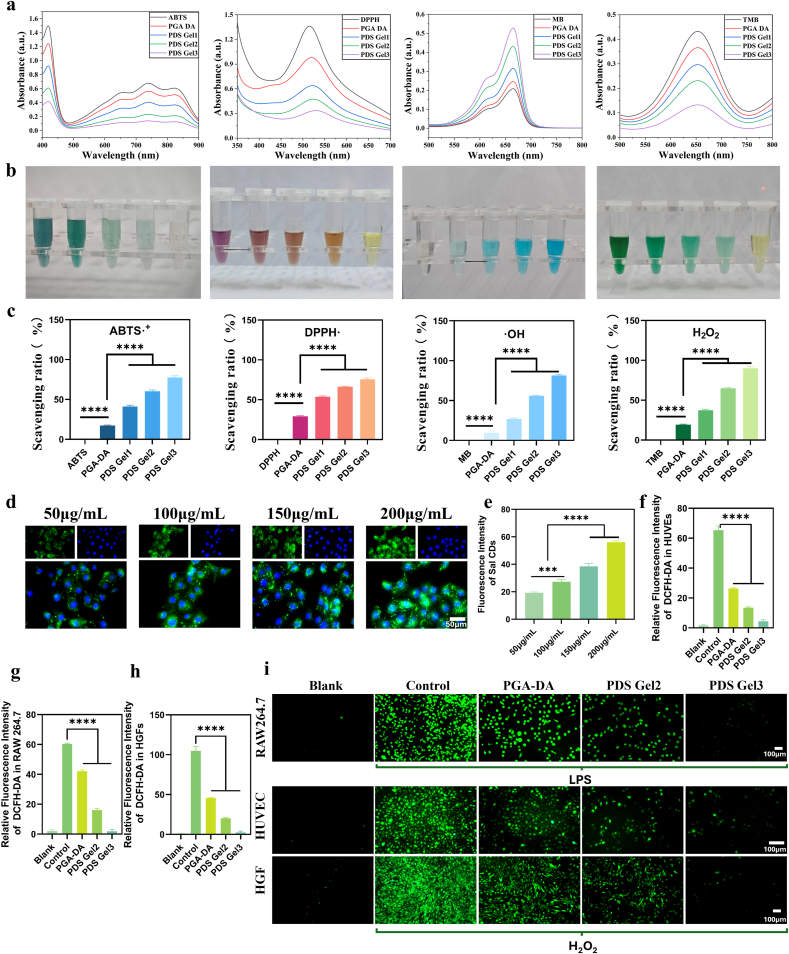
**ROS scavenging ability and intracellular antioxidant activity.** (a) ROS scavenging activities of different treatments evaluated by UV–vis spectroscopy, including DPPH•, ABTS**•^+^**·, •OH radicals, and H_2_O_2_, with corresponding absorption spectra. (b) Photographs showing the color changes of DPPH•, ABTS**•^+^**·, •OH, and H_2_O_2_ solutions after treatment with different samples. (c) Quantitative analysis of the scavenging efficiencies toward DPPH•, ABTS**•^+^**·, •OH radicals, and H_2_O_2_ based on UV–vis absorption data. (d) Fluorescence microscopy images showing the uptake of Sal-CDs by HUVECs at different concentrations (50, 100, 150, and 200 μg mL^−1^). (e) Quantitative analysis of intracellular fluorescence intensity corresponding to (d). (f–h) Quantitative analysis of intracellular ROS fluorescence intensity in HUVECs, HGFs, and RAW264.7 cells corresponding to (i). (i) Fluorescence microscopy images of intracellular ROS levels in HUVECs, HGFs, and RAW264.7 cells detected using the DCFH-DA probe. Data are presented as mean ± SD (n = 3). Statistical analysis was performed using one-way ANOVA followed by Tukey's post hoc test. ∗*P* < 0.05, ∗∗*P* < 0.01, ∗∗∗*P* < 0.001, and ∗∗∗∗*P* < 0.0001. (For interpretation of the references to color in this figure legend, the reader is referred to the Web version of this article.)

In vitro chemical assays, including ABTS•^+^ and DPPH• radical scavenging, Fenton reaction–mediated MB degradation, and the HRP/H_2_O_2_/TMB enzymatic colorimetric system, were employed to systematically evaluate the capacity of PDS Gel to regulate various free radicals and ROS. Compared with a control solution lacking any antioxidant components, PGA-DA hydrogel itself exhibited a certain degree of radical scavenging and oxidative inhibition, likely attributable to the catechol moieties retained from dopamine within the polymer network. Previous studies have shown that catechol groups can participate in oxidative stress modulation via hydrogen donation, electron transfer, or metal ion chelation, thereby endowing dopamine-containing materials with intrinsic antioxidant potential [19,42,43]. Building on this, the incorporation of Sal-CDs further enhanced the ability of PDS Gel to scavenge or inhibit various free radicals and ROS. UV–visible spectroscopy showed that PDS Gel loaded with Sal-CDs markedly reduced the characteristic absorption peaks of ABTS•^+^, DPPH•, and •OH, with the effect increasing progressively with higher Sal-CDs content, demonstrating clear concentration dependence (Fig. 4a–c). Specifically, PDS Gel significantly inhibited •OH-mediated methylene blue degradation in the Fenton reaction and effectively reduced TMB color development in the HRP/H_2_O_2_/TMB system, indicating that the hydrogel can not only scavenge free radicals but also modulate physiologically relevant ROS such as •OH and H_2_O_2_. These results suggest a synergistic effect between the intrinsic antioxidant capacity of the PGA-DA matrix and the introduced Sal-CDs, endowing the hydrogel system with multi-pathway potential for regulating oxidative stress.

Since in vitro chemical assays primarily reflect the upper limit of a material's response to oxidative reactions, their biological effects still depend largely on the accessibility of functional components within the cellular environment. Fluorescence imaging demonstrated that Sal-CDs were effectively internalized by cells and distributed intracellularly, providing a necessary basis for regulating oxidative stress, maintaining redox homeostasis, and exerting subsequent physiological functions at the cellular level (Fig. 4d and e). Considering biocompatibility, antibacterial and antioxidant performance, and cellular accessibility, PDS Gel2 and PDS Gel3 were selected for subsequent experiments.

In oxidative stress models using HUVECs, RAW264.7 cells, and HGFs, the unstimulated Blank group exhibited the lowest intracellular ROS levels. Treatment with PGA-DA or PDS Gel significantly reduced intracellular ROS in a manner dependent on Sal-CDs content, although levels remained higher than those in the Blank group. These findings indicate that PDS Gel does not completely eliminate intracellular ROS but likely acts by inhibiting excessive accumulation and limiting over-oxidation, thereby helping cells restore a relatively balanced redox state (Fig. 4f–i). This “buffering” rather than “eradication” mode of ROS regulation aligns with the physiological role of ROS as signaling molecules in normal cellular function.

In summary, PDS Gel demonstrates stable ROS-regulating capacity in vitro chemical assays through the synergistic contributions of the PGA-DA matrix and Sal-CDs. Sal-CDs exhibit good cellular accessibility and effectively alleviate intracellular oxidative stress under inflammatory stimulation. Together, these results systematically validate the hydrogel's potential for oxidative stress modulation from the perspectives of chemical reactivity, biological accessibility, and cellular functional response, providing a mechanistic foundation for its subsequent immunoregulatory and tissue-repair–promoting functions.

### 3.5 Modulation of macrophage polarization by the material

Given that ROS are critical amplifiers of inflammatory cascades, we hypothesized that the ROS-scavenging ability of PDS Gel would modulate macrophage behavior. Chronic inflammatory responses are a key factor in delayed wound healing and abnormal tissue repair, with macrophages playing a central role in regulating the immune microenvironment of the wound. Classically activated M1 macrophages secrete large amounts of pro-inflammatory cytokines, exacerbating tissue damage, whereas alternatively activated M2 macrophages promote inflammation resolution and tissue regeneration [44]. Therefore, regulating macrophage polarization is considered a key strategy for achieving high-quality wound healing [45]. To systematically evaluate the regulatory effect of PDS Gel on M1 macrophage polarization under inflammatory conditions, LPS-stimulated RAW264.7 cells were used as an in vitro model. The following groups were established: a blank control group (Blank), a model control group (Control), and treatment groups treated with PGA-DA, PDS Gel2, and PDS Gel3, respectively, following LPS stimulation. Systematic analysis of M1 macrophage-associated markers was performed across all groups.

**Fig. 5:**
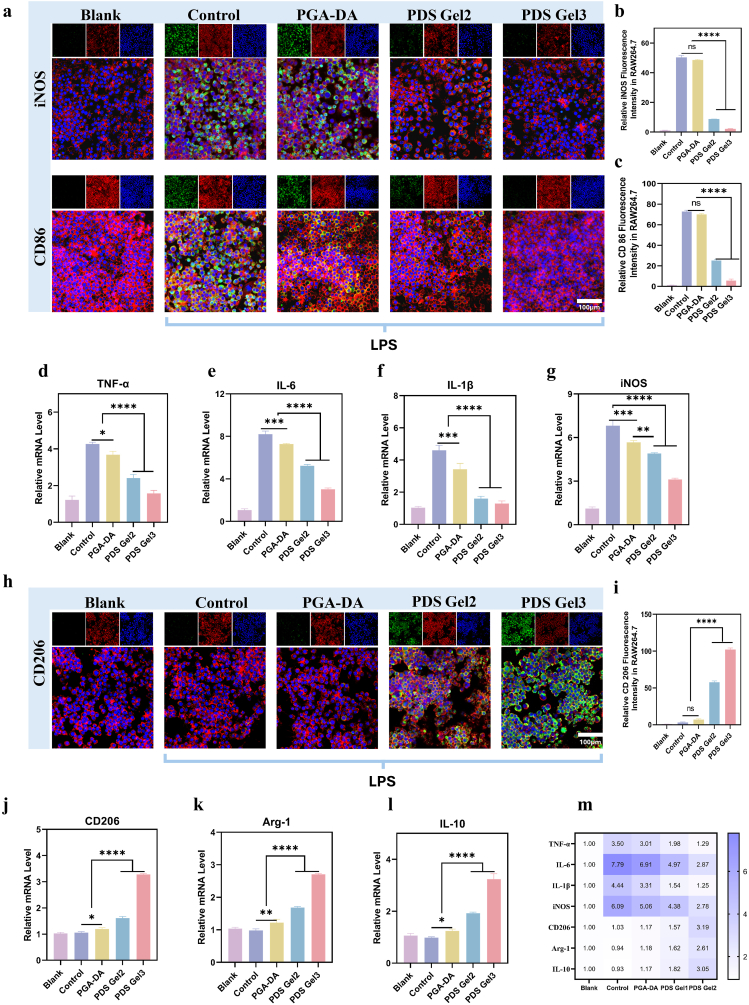
**Regulation of macrophage polarization by PDS Gel.** (a) IF images of M1 markers (iNOS and CD86) in RAW264.7 cells. (b, c) Quantitative analysis of fluorescence intensity corresponding to (a). (d–g) qPCR analysis of M1-related mRNA expression levels in RAW264.7 cells after different treatments. (h) IF images of the M2 marker (CD206) in RAW264.7 cells. (i) Quantitative analysis of CD206 fluorescence intensity corresponding to (h). (j–l) qPCR analysis of M2-related mRNA expression levels in RAW264.7 cells after different treatments. (m) Heatmap of mRNA expression levels in RAW264.7 cells. Data are presented as mean ± SD (n = 3). Statistical analysis was performed using one-way ANOVA followed by Tukey's post hoc test. ∗*P* < 0.05, ∗∗*P* < 0.01, ∗∗∗*P* < 0.001, and ∗∗∗∗*P* < 0.0001.

In the LPS-induced inflammatory model, macrophages in the Control group exhibited typical M1 polarization, with IF showing significantly enhanced CD86 and iNOS expression. In contrast, PGA-DA treatment resulted in no significant changes in the fluorescence intensity of CD86 and iNOS. On this basis, Sal-CDs-loaded PDS Gel2 and PDS Gel3 further markedly suppressed the expression of M1 phenotypic markers in a dose-dependent manner, with PDS Gel3 showing the most pronounced inhibitory effect (Fig. 5a–c). The ELISA results further validated the above trend at the protein level, showing that iNOS was significantly reduced in the PDS Gel–treated group (Fig. S10). qPCR results were highly consistent with the IF findings, demonstrating that PDS Gel treatment significantly downregulated the transcriptional levels of iNOS, TNF-α, IL-6, and IL-1β (Fig. 5d–g), suggesting effective attenuation of the pro-inflammatory cytokine cascade. These results indicate that PDS Gel effectively inhibits excessive macrophage polarization toward the pro-inflammatory M1 phenotype under inflammatory stimulation and reduces the release of inflammatory factors. Mechanistically, this inhibitory effect may be closely associated with the material's modulation of the inflammation-related oxidative stress microenvironment, thereby influencing the activation state of downstream inflammatory signaling pathways.

In addition to inhibiting M1 polarization, PDS Gel also significantly promoted macrophage polarization toward the M2 phenotype. IF results showed that, compared with the Control group, CD206 expression was not markedly upregulated in the PGA-DA group, whereas treatment with PDS Gel2 and PDS Gel3 led to a significant enhancement of the CD206 fluorescence signal, with the most pronounced effect in the PDS Gel3 group (Fig. 5h and i). The ELISA results also supported this trend, showing that M2-associated functional markers were upregulated after PDS Gel treatment (Fig. S10). qPCR analysis further confirmed this trend. Compared with the Control group, PDS Gel treatment significantly upregulated the gene expression levels of Arg-1, CD206, and IL-10 in a manner that intensified with increasing Sal-CDs content (Fig. 5j–m). These findings indicate that PDS Gel not only suppresses pro-inflammatory responses but also actively induces the M2 macrophage phenotype, which is associated with immunomodulatory and tissue repair functions, thereby promoting the transition from the inflammatory phase to the resolution and repair phase.

Notably, PGA-DA treatment induced modest transcriptional changes in both M1-and M2-associated genes; however, the corresponding IF signals showed no significant variation. This suggests that the pure hydrogel system exerts a relatively mild influence on macrophage polarization, likely mitigating inflammatory and oxidative stress microenvironments rather than directly driving a definitive M1 or M2 phenotypic transition. This effect may be associated with the intrinsic redox-regulatory capacity conferred by the dopamine structure, although its magnitude appears limited. These findings further highlight the critical role of Sal-CDs incorporation in achieving pronounced immunophenotypic regulation.

Combined with the previously observed ROS regulation, it is inferred that PDS Gel does not exert its anti-inflammatory effect by completely eliminating intracellular ROS; rather, it restores redox homeostasis, thereby modulating the amplification of inflammatory signaling and creating a more favorable microenvironment for M2 polarization. Overall, this coordinated “anti-inflammatory–pro-regenerative” regulation suggests that the material may optimize the immune microenvironment through systemic remodeling of inflammation-related molecular networks. To further elucidate the underlying molecular mechanisms, transcriptomic (RNA-seq) analysis was subsequently performed.

### 3.6 Transcriptomic (RNA-seq) analysis

**Fig. 6:**
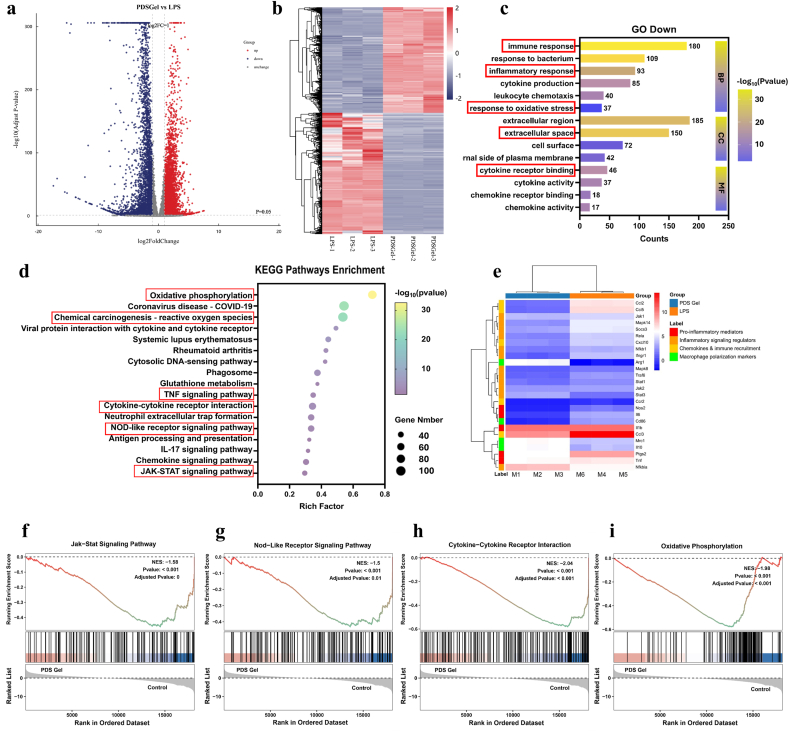
**Transcriptomic analysis of macrophages.** (a) Volcano plot of differentially expressed genes (DEGs). (b) Heatmap of DEGs. (c) Downregulated pathways identified by Gene Ontology (GO) enrichment analysis. (d) Downregulated pathways identified by KEGG pathway analysis. (e) Heatmap of significantly altered genes in KEGG-enriched pathways. (f) GSEA of the JAK–STAT signaling pathway. (g) GSEA of the NOD-like receptor signaling pathway. (h) GSEA of the cytokine–cytokine receptor interaction pathway. (i) GSEA of the oxidative phosphorylation pathway.

To elucidate the immunomodulatory mechanism of PDS Gel, RNA-seq was performed on LPS-stimulated macrophages with or without PDS Gel treatment. As shown in Fig. 6a and b, a total of 8435 differentially expressed genes (DEGs) were identified, including 3917 upregulated and 4518 downregulated genes, indicating that PDS Gel markedly reshaped the transcriptional landscape of inflammatory macrophages.

GO and KEGG enrichment analyses (Fig. 6c and d) revealed that the downregulated genes were mainly associated with inflammation-related biological processes, including inflammatory response, cytokine production, and immune cell chemotaxis, and were significantly enriched in classical inflammatory signaling pathways such as the TNF, JAK–STAT, and NOD-like receptor pathways. At the gene level (Fig. 6e), key pro-inflammatory mediators including *Tnf*, *Il1b*, *Il6*, and *Nos2* were significantly downregulated. Meanwhile, the expression of core regulators of inflammatory signaling, such as *Nfkb1*, *Rela*, *Mapk14*, *Jak2*, and *Stat3*, was also reduced, suggesting suppression of the amplification of inflammatory signaling cascades.

Notably, pathway analysis also revealed a significant downregulation of the oxidative phosphorylation pathway, accompanied by the suppression of oxidative stress-related processes. Considering that inflammatory activation of macrophages is often associated with excessive mitochondrial metabolic activation and increased ROS production, these results suggest that PDS Gel alleviates metabolic overactivation and oxidative stress at the transcriptional level, thereby disrupting the “inflammation–metabolism” positive feedback loop. Consistently, macrophage polarization-related genes also showed marked changes: the expression of M1-associated markers (*Nos2* and *Cd86*) decreased, whereas M2-associated genes (Arg*1*, *Mrc1*) and the anti-inflammatory cytokine *Il10* were upregulated, indicating a phenotypic shift of macrophages toward a pro-repair M2 phenotype. Further gene set enrichment analysis (GSEA) (Fig. 6f–i) confirmed the overall downregulation of inflammatory signaling pathways and oxidative metabolic processes at the gene-set level, consistent with the DEG enrichment results. In contrast, upregulated genes were mainly involved in DNA repair and cellular homeostasis-related processes (Fig. S12), suggesting that cells gradually restored homeostasis following the alleviation of inflammatory and metabolic stress.

Collectively, PDS Gel coordinately suppressed inflammatory signaling pathways, reduced chemokine-associated signaling, and alleviated oxidative stress while activating cellular repair and homeostasis-maintaining programs, thereby globally reshaping the transcriptional profile of inflammatory macrophages. These findings provide important molecular insights into the ability of PDS Gel to promote inflammation resolution and accelerate tissue repair.

### 3.7 Pro-angiogenic and cell Migration–Promoting effects

The transcriptomic data revealed downregulation of inflammatory pathways and upregulation of repair-related genes; we therefore investigated whether these molecular changes would enhance angiogenesis. Angiogenesis is a key biological event in wound healing, enabling nutrient transport and the reconstruction of newly formed tissue. During this process, endothelial cell migration, tube-forming capacity, and responsiveness to pro-angiogenic signals play pivotal roles in neovascularization. Using HUVECs as the model, two baseline groups were established: an unstimulated Blank group and an LPS-induced inflammatory model group (Control). Under inflammatory stimulation, additional treatment groups were introduced, in which cells were treated with PGA-DA, PDS Gel2, or PDS Gel3, respectively, to evaluate the regulatory effects of these materials on endothelial angiogenic behaviors. The angiogenic potential of each group in the inflammatory microenvironment was systematically assessed through cell migration assays, in vitro tube formation assays, and analysis of VEGF expression.

**Fig. 7:**
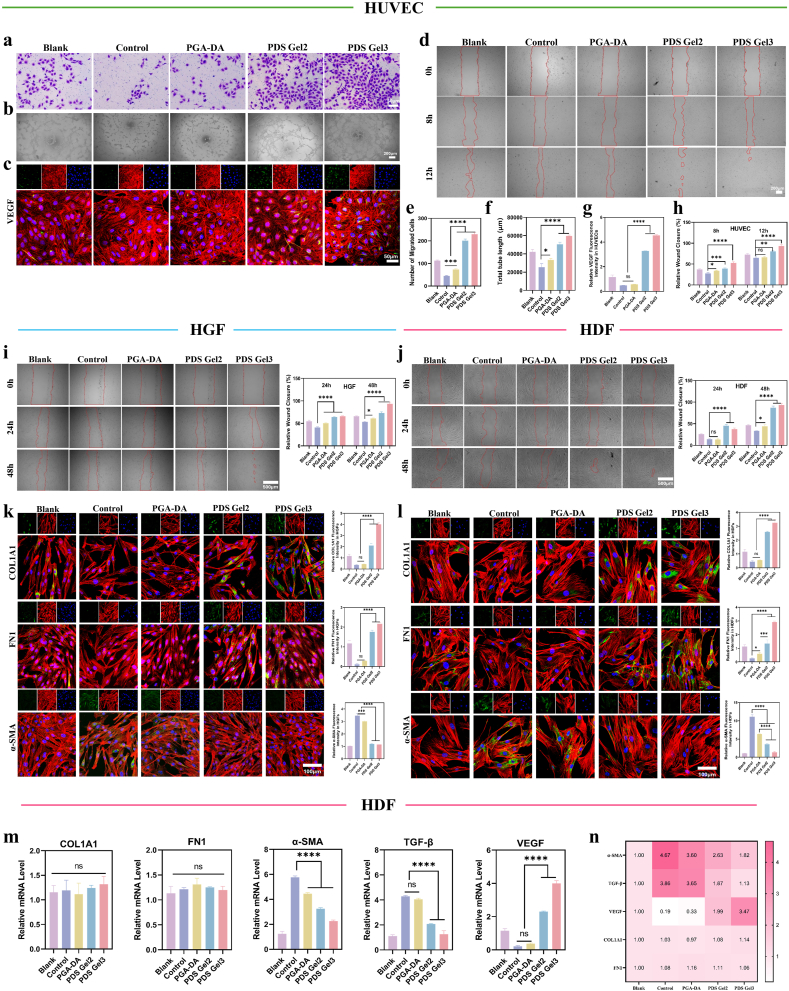
**Regulation of angiogenesis and ECM remodeling in fibroblasts by different treatments.** (a) Migration of HUVECs under different treatments. (b) Tube formation of HUVECs after treatment. (c) VEGF IF in HUVECs. (d) Scratch assay of HUVECs, with red circles indicating the initial wound area. (e–h) Quantitative analyses corresponding to (a–d), including (e) number of migrated HUVECs, (f) total tube length in tube formation assay, (g) VEGF fluorescence intensity, and (h) relative wound closure rate (%), calculated as $\text{Relative}\;\text{closure}=\frac{\text{Initial}\;\text{area}\;‐\;\text{Current}\;\text{area}}{\text{Initial}\;\text{area}}\times 100\%$. (i–j) Migration of HGFs (i) and HDFs (j) under different treatments, with statistical quantification. (k–l) IF and quantification of ECM components COL1A1, FN1, and myofibroblast marker α-SMA in HGFs (k) and HDFs (l). (m) qPCR analysis of ECM deposition- and myofibroblast activation-related genes in HDFs after different treatments. (n) Heatmap showing mRNA expression levels of the corresponding genes in HDFs. Data are presented as mean ± SD (n = 3). Statistical analysis was performed using one-way ANOVA followed by Tukey's post hoc test. ∗*P* < 0.05, ∗∗*P* < 0.01, ∗∗∗*P* < 0.001, and ∗∗∗∗*P* < 0.0001. (For interpretation of the references to color in this figure legend, the reader is referred to the Web version of this article.)

Under the LPS-induced inflammatory microenvironment, the migratory capacity of HUVECs in the Control group was markedly inhibited, as evidenced by a significant reduction in the number of migrated cells in the Transwell assay (Fig. 7a and e). In contrast, treatment with PGA-DA partially restored endothelial cell migration, showing an increased number of migrated cells compared with the Control group, suggesting that this hydrogel system may alleviate inflammation-induced impairment of endothelial migration by improving cell adhesion and stabilizing the local microenvironment.

On this basis, PDS Gel2 and PDS Gel3, loaded with Sal-CDs, further significantly enhanced HUVEC migration, with the number of migrated cells increasing progressively with higher Sal-CDs loading, among which PDS Gel3 exhibited the most pronounced effect. Consistent with the Transwell results, the scratch assay also showed the lowest wound closure rate in the Control group (Fig. 7d and h). PGA-DA moderately promoted closure of the scratched area, whereas PDS Gel2 and PDS Gel3 markedly accelerated wound closure, with the closure rate increasing in a Sal-CDs dose-dependent manner; again, PDS Gel3 demonstrated the fastest closure.

These results indicate that PDS Gel not only improves the survival and adhesion microenvironment of endothelial cells under inflammatory conditions but may also provide a more favorable milieu for endothelial migration by modulating inflammatory responses and oxidative stress–related processes, thereby promoting angiogenesis-related events.

The effects of the materials on angiogenic behavior were further evaluated using an in vitro tube formation assay. As shown in Fig. 7b and f, HUVECs in the Control group failed to form continuous and stable tubular structures, displaying poor network integrity. After PGA-DA treatment, tube-forming ability was partially improved; however, the resulting structures remained relatively scattered with limited network complexity, suggesting a modest pro-angiogenic effect. In contrast, treatment with PDS Gel2 and PDS Gel3 enabled HUVECs to rapidly assemble into clear and continuous capillary-like networks, with PDS Gel3 exhibiting the greatest tube length and the most well-developed network morphology. These findings indicate that the incorporation of Sal-CDs confers a clear advantage to the material system in supporting endothelial angiogenic behavior.

Notably, although PGA-DA showed certain positive effects in migration and tube formation assays, its overall promotion remained limited. This effect may primarily arise from passive regulation through improved physical support and stabilization of the local microenvironment. Due to the lack of active modulation of angiogenesis-related signaling pathways, its ability to enhance the angiogenic potential of endothelial cells appears constrained.

To further explore the potential mechanism underlying the pro-angiogenic effects of PDS Gel, the IF expression of VEGF in HUVECs was analyzed. As shown in Fig. 7c and g, VEGF fluorescence intensity was markedly enhanced in the PDS Gel2 and PDS Gel3 groups compared with the Control group, exhibiting a gradual increase with higher Sal-CDs content. Consistently, the VEGF levels measured by ELISA were in good agreement with the IF results (Fig. S11). The elevated VEGF expression suggests a more active pro-angiogenic phenotype of endothelial cells after material treatment, consistent with the results of the migration and tube formation assays. Together, these findings suggest that PDS Gel may enhance endothelial angiogenic behavior by improving the inflammation- and oxidative stress–related microenvironment.

Overall, PDS Gel significantly promotes HUVEC migration and in vitro tube formation under inflammatory conditions and enhances cellular responsiveness to angiogenesis-related signals. In comparison, PGA-DA can moderately improve endothelial cell survival and basal functional status, but its regulatory effect remains relatively mild, further highlighting the critical role of Sal-CDs in constructing a microenvironment favorable for angiogenesis. Combined with the previously demonstrated anti-inflammatory and immunomodulatory effects, PDS Gel regulates the inflammation–immunity–angiogenesis axis at multiple levels, thereby supporting vascular regeneration and subsequent tissue remodeling during wound healing.

### 3.8 Effects on ECM protein expression and gene regulation in fibroblasts

This study aims to develop a functional hydrogel with potential for wound repair. Fibroblasts play essential roles during wound healing, including migration, ECM synthesis, and tissue remodeling, and their behavior directly influences the quality of tissue repair [46]. Therefore, HGFs and HDFs were employed to systematically evaluate the regulatory effects of PDS Gel on fibroblast migration and fibrosis-related phenotypes under an inflammatory microenvironment.

Under inflammatory stimulation, the migration of both HGFs and HDFs was markedly impaired, with significantly slower scratch closure, indicating that inflammatory signals disrupt fibroblast migration during the early phase of wound repair. Treatment with PDS Gel substantially improved migration and accelerated scratch closure in both cell types (Fig. 7i and j), suggesting that the hydrogel can restore fibroblast migratory potential under inflammatory conditions, providing the necessary cellular dynamics for effective wound healing.

Fibroblast ECM expression and fibrosis-related phenotypes were further evaluated by IF. α-SMA is a key marker of fibroblast-to-myofibroblast differentiation [47], with elevated levels indicating an “activated” state, whereas COL1A1 and FN1 are major ECM components [48], reflecting matrix synthesis and repair capacity. Under inflammatory conditions, α-SMA expression was markedly increased, while COL1A1 and FN1 were decreased, indicating an inflammation-induced activated phenotype with impaired ECM synthesis [[49], [50], [51], [52]]. PDS Gel treatment restored COL1A1 and FN1 expression to appropriate levels and moderately reduced α-SMA, preserving partial myofibroblast activity (Fig. 7k and l). This suggests that the hydrogel mitigates excessive inflammation-induced activation without fully suppressing the myofibroblast phenotype, maintaining fibroblast reparative potential.

At the transcriptional level, qPCR further confirmed these trends. Given the representativeness of HDFs in wound repair and angiogenic microenvironment regulation, HDFs were used to assess key inflammation- and angiogenesis-related genes [53,54]. Inflammatory stimulation significantly upregulated α-SMA and the profibrotic factor TGF-β [[55], [56], [57]], whereas transcription of ECM components COL1A1 and FN1 showed no significant change. This suggests that LPS-induced fibroblast activation primarily affects α-SMA/TGF-β transcription, while reduced ECM synthesis occurs at the translational or post-translational level (e.g., protein degradation), rather than via direct gene repression [58,59]. PDS Gel effectively suppressed excessive α-SMA and TGF-β1 upregulation while maintaining COL1A1 and FN1 transcription at baseline. Coupled with the observed restoration of COL1A1 and FN1 protein expression, this “transcriptional stability–protein recovery” pattern indicates that the hydrogel restores normal ECM synthesis by improving the microenvironment rather than driving gene overexpression, while concurrently blocking pathological inflammatory activation. This precise, mild regulation avoids excessive fibrosis and promotes functional ECM reconstruction.

Additionally, VEGF mRNA was moderately upregulated [60], suggesting that the hydrogel not only relieves inflammation-induced fibroblast dysfunction but also provides pro-angiogenic signals favorable for subsequent vascularization and tissue repair (Fig. 7m and n).

Notably, under inflammatory conditions, PGA-DA had limited effects on fibroblast migration and ECM markers, whereas the Sal-CDs–loaded PDS Gel exhibited more pronounced, integrated regulation: promoting cell migration, restoring ECM synthesis to physiological levels, and modulating α-SMA and TGF-β1 expression. These results highlight the critical role of PDS Gel in improving the inflammatory microenvironment and regulating fibroblast behavior, providing a cellular and molecular basis for subsequent in vivo wound repair and tissue reconstruction studies.

### 3.9 In vivo wound healing evaluation

Following the systematic evaluation of PDS Gel's in vitro bioactivity, a critical question remained: whether its multidimensional functional advantages could translate into effective wound healing within the complex in vivo microenvironment. Specifically, we asked whether the observed anti-inflammatory polarization, enhanced angiogenesis, and fibroblast-mediated ECM remodeling would collectively accelerate wound closure. To comprehensively assess the therapeutic potential of this composite material, two representative wound models were employed in parallel: a rat recurrent oral mucosal ulcer model and a full-thickness skin defect model. Through histological analysis, molecular biology assays, and quantitative morphological evaluation, the in vivo effects of PDS Gel on wound closure, inflammation modulation, and tissue regeneration were systematically investigated.

#### 3.9.1 Oral ulcer model

The mucoadhesive performance of therapeutic materials is critical for the treatment of recurrent oral ulcers, as strong adhesion in the complex oral environment ensures effective lesion protection and drug delivery. To evaluate this property under dynamic oral conditions, we assessed the retention behavior of PDS Gel on rat tongue (Fig. S13). The hydrogel exhibited strong initial adhesion, with intense fluorescence signals observed within the first 1 h, followed by a moderate retention rate between 1 and 1.5 h. Thereafter, the signal decreased markedly, with only sparse punctate residues remaining between 1.5 and 2 h, indicating gradual detachment under physiological oral dynamics. Consistently, adhesion durability tests in a porcine mucosal model revealed stable performance across different oral pH conditions, with average failure times of 2.2 h (pH 5.5), 1.97 h (pH 6.5), and 1.78 h (pH 7.4) (Fig. S14). Together, these results demonstrate that PDS Gel can maintain effective mucosal coverage for approximately 1.5–2 h in dynamic oral environments. This duration is clinically meaningful as it provides sufficient protection of the lesion surface during the critical early stage after each application and enables initial rapid drug release. Based on this favorable local retention ability, the therapeutic efficacy of PDS Gel in oral ulcer healing was further investigated. Considering the above experimental results, PDS Gel3 was selected for subsequent in vivo experiments.

**Fig. 8:**
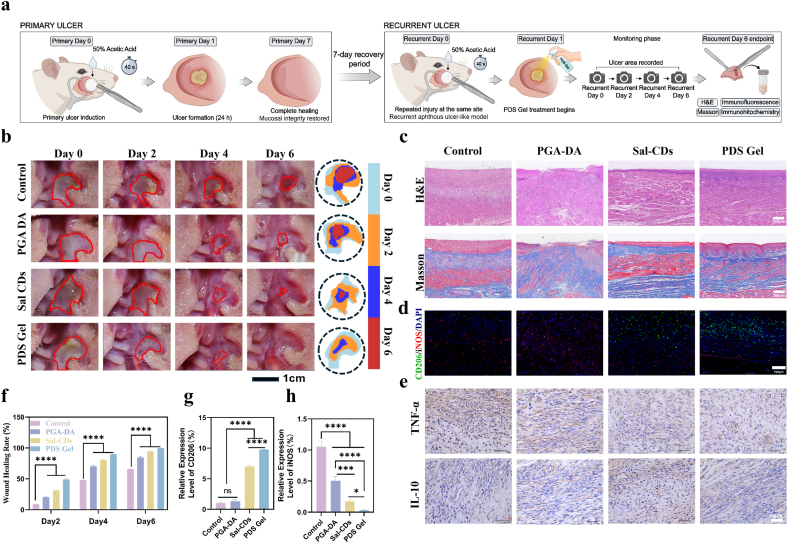
**Evaluation of the therapeutic effects of PDS Gel in SD rat oral ulcer model.** (a) Schematic illustration of oral ulcer induction, treatment, and wound healing in different groups. (b) Representative images of ulcer healing and schematic progression over time. (c) HE and Masson staining of wound sites after different treatments. (d) IF images of CD206 and iNOS in wound tissues. (e) Immunohistochemical staining of TNF-α and IL-10 in wound tissues after different treatments. (f) Quantification of wound closure rates corresponding to (b). (g–h) Quantitative analysis of CD206 and iNOS fluorescence corresponding to (d). Data are presented as mean ± SD (n = 6). Statistical analysis was performed using one-way ANOVA followed by Tukey's post hoc test. ∗*P* < 0.05, ∗∗*P* < 0.01, ∗∗∗*P* < 0.001, and ∗∗∗∗*P* < 0.0001.

First, in the recurrent oral mucosal ulcer model, wound images were recorded on days 0, 2, 4, and 6 after modeling, and ulcer area changes were quantitatively analyzed (Fig. 8a). As shown in Fig. 8b, wound areas in all groups gradually decreased over time, but the healing rates differed markedly. Quantitative analysis (Fig. 8f) revealed that by day 6 the Control group still exhibited a relatively large ulcer area, whereas the PGA-DA and Sal-CDs groups showed partial improvement. In contrast, the PDS Gel group displayed the most pronounced wound contraction, demonstrating the best therapeutic effect in promoting ulcer healing.

To further evaluate tissue regeneration quality, H&E and Masson's trichrome staining were performed on day-6 tissues (Fig. 8c). In the PDS Gel group, a continuous stratified squamous epithelium was formed, with well-organized epithelial cells and clearly defined basal, spinous, and keratinized layers. Regular epithelial rete ridges were observed, and the tissue architecture closely resembled normal oral mucosa. Inflammatory infiltration beneath the epithelium was reduced, and the connective tissue structure was intact. Masson staining showed abundant and well-aligned collagen fibers with a clear boundary from the underlying muscle, indicating effective epithelial–connective tissue interface reconstruction. The Sal-CDs group also exhibited continuous epithelial coverage, although the epithelial layer was thinner and rete ridges were less pronounced than in the PDS Gel group. H&E staining showed orderly muscle fibers beneath the epithelium, while Masson staining revealed relatively abundant but slightly irregular collagen deposition accompanied by some neovascularization. In the PGA-DA group, repair was slower, with incomplete epithelial coverage, absence of rete ridges, and notable inflammatory infiltration with evident granulation tissue. Masson staining showed dense but disorganized collagen fibers with unclear boundaries from surrounding muscle. The Control group showed the most delayed healing, characterized by minimal epithelial coverage, necrotic tissue and exudate at the wound surface, marked inflammatory infiltration, and sparse, uneven collagen distribution. Overall, macroscopic observations and histological analysis consistently demonstrate that PDS Gel significantly accelerates oral ulcer healing while promoting complete epithelial reconstruction and orderly collagen remodeling.

To further investigate the immunomodulatory effects of PDS Gel, macrophage polarization was evaluated by immunofluorescent co-staining of the M1 marker iNOS and M2 marker CD206 in ulcer tissues (Fig. 8d,g,h). Distinct polarization patterns were observed among groups. The PDS Gel group exhibited the strongest CD206 signal with relatively low iNOS expression, whereas the Sal-CDs group showed moderate CD206 expression accompanied by detectable iNOS signals. In contrast, the PGA-DA and Control groups displayed strong iNOS expression but weak CD206 signals. These results indicate that PDS Gel effectively regulates macrophage polarization, promoting the transition from the pro-inflammatory M1 phenotype to the anti-inflammatory M2 phenotype, thereby suppressing excessive inflammation and facilitating repair-associated immune regulation.

Inflammation-related cytokines TNF-α and IL-10 were further analyzed to assess the inflammatory microenvironment. TNF-α is a classical pro-inflammatory cytokine involved in initiating and amplifying inflammatory responses, whereas IL-10 is a key anti-inflammatory mediator that suppresses excessive inflammation and promotes the transition toward tissue repair. Immunohistochemical staining revealed distinct expression patterns across treatment groups (Fig. 8e and S15). TNF-α signals were mainly localized in inflammatory and stromal cells within the wound tissue, with the strongest expression in the Control group, slightly reduced levels in the PGA-DA group, further decreased levels in the Sal-CDs group, and the weakest expression in the PDS Gel group, indicating effective attenuation of local inflammation. Conversely, IL-10 expression showed a gradual increase across groups: low in the Control group, moderately elevated in the PGA-DA and Sal-CDs groups, and highest in the PDS Gel group, where IL-10 signals were broadly distributed throughout the wound stroma. Collectively, the downregulation of TNF-α and upregulation of IL-10 in the PDS Gel group suggest that this material effectively modulates the inflammatory microenvironment, facilitating the transition from inflammation to tissue repair.

Overall, owing to its excellent mucoadhesive properties, PDS Gel significantly promotes the healing of oral ulcers. It not only accelerates wound closure and enhances epithelial regeneration and collagen remodeling, but also reshapes the inflammatory microenvironment toward a pro-healing state.

#### 3.9.2 Skin defect model

**Fig. 9:**
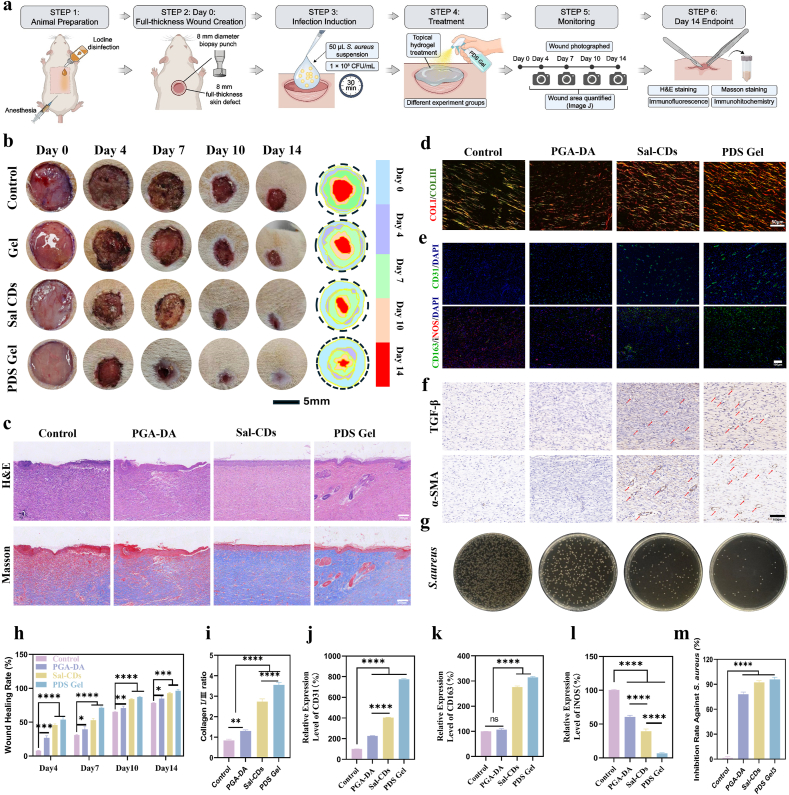
**Evaluation of the therapeutic effects of PDS Gel in SD rat skin defect model.** (a) Schematic illustration of skin defect modeling in rats. (b) Representative images of wound healing and schematic progression over time. (c) HE and Masson staining of wound sites after different treatments. (d) Sirius Red staining showing collagen deposition. (e) IF images of CD31, CD163, and iNOS in wound tissues. (f) Immunohistochemical staining of TGF-β and α-SMA in wound tissues. (g) Representative images of bacterial colonies on agar plates after treatment. (h) Quantification of wound closure rates corresponding to (b). (i) Ratio of type I to type III collagen from Sirius Red staining, with normal skin ratio ∼4. (j–l) Quantitative analyses of CD31, CD163, and iNOS fluorescence corresponding to (e). (m) Relative antibacterial rate quantified from (g). Data are presented as mean ± SD (n = 6). Statistical analysis was performed using one-way ANOVA followed by Tukey's post hoc test. ∗*P* < 0.05, ∗∗*P* < 0.01, ∗∗∗*P* < 0.001, and ∗∗∗∗*P* < 0.0001. (For interpretation of the references to color in this figure legend, the reader is referred to the Web version of this article.)

To further evaluate the therapeutic efficacy of PDS Gel in a complex infected microenvironment, a rat full-thickness infected wound model was established. Wound healing was continuously documented by photography on days 0, 4, 7, 10, and 14 after modeling (Fig. 9a). Macroscopic observation showed that wounds in all groups gradually contracted over time, but the healing rates differed significantly (Fig. 9b and h). Among the groups, the PDS Gel–treated wounds exhibited the fastest and most pronounced contraction, indicating that this composite hydrogel can effectively accelerate the closure of infected wounds and enhance the overall healing process.

To further assess the quality of tissue regeneration, wound tissues on day 14 were analyzed by H&E and Masson's trichrome staining (Fig. 9c). Histological examination revealed that, in the Control group, the epidermal structure was discontinuous, dermal cells were disorganized, and there was prominent inflammatory cell infiltration. Masson staining showed sparse and uneven collagen fiber distribution, indicating that the wounds remained in the inflammatory and early proliferative phases. After PGA-DA treatment, epidermal continuity was partially restored, but repair was still dominated by a filling-type pattern, with collagen deposition showing some disorder. The Sal-CDs group further alleviated inflammation and improved collagen deposition, resulting in a more uniform dermal structure; however, skin appendage structures remained poorly developed, suggesting that tissue was still in an active proliferative stage. In contrast, the PDS Gel group exhibited features of more mature tissue reconstruction. H&E staining showed a continuous, well-layered epidermis and orderly dermal organization, with observable hair follicle–like structures. Masson staining revealed uniform and well-aligned collagen fibers, closely resembling normal skin architecture. These findings indicate that PDS Gel not only accelerates wound closure but also promotes higher-quality tissue regeneration.

The composition and maturation of collagen in regenerated tissue were assessed using Sirius Red staining under polarized light (Fig. 9d and i). Collagen fibers exhibited characteristic birefringence: green signals corresponded to thinner type III collagen, whereas red/orange signals indicated thicker type I collagen. During early healing, collagen fibers were predominantly green, reflecting the presence of immature type III collagen within the newly formed granulation tissue. As wound healing progressed, the proportion of red birefringence increased, and collagen fibers became thicker and more regularly aligned. Quantitative analysis (Fig. 9i) confirmed a significantly optimized type I/III collagen ratio in the PDS Gel group, which is a hallmark of high-quality ECM remodeling. This balanced transition is closely associated with the biomechanical potential of regenerated mucosa; specifically, the initial type III collagen provides necessary flexibility, while the subsequent increase in type I collagen enhances tensile strength and tissue compliance. The orderly and dense alignment of these fibers, as opposed to the disorganized deposition in the control group, indicates that the PDS Gel promotes a more functional and mature ECM architecture, thereby minimizing the risk of fibrotic scarring. To further investigate the potential mechanisms of action, wound tissues were analyzed by IF staining. In the PDS Gel–treated group, CD31 expression was enhanced, indicating more active neovascularization (Fig. 9e and j). Notably, this high-quality ECM reconstruction is mechanistically linked to the coordinated microenvironment transition. IF staining showed that the PDS Gel promoted M2 macrophage polarization (Fig. 9e,k,l), consistent with the immunomodulatory effects observed in Fig. 8d. This pro-healing microenvironment further promoted the moderate and spatially organized activation of TGF-β signaling (Fig. 9f and S16) to guide downstream matrix synthesis. By maintaining a localized and balanced expression of TGF-β1 and α-SMA, the PDS Gel effectively prevents excessive myofibroblast activation and collagen over-production. This expression pattern indicates that the wound tissue has transitioned from the proliferative phase toward the remodeling phase, with myofibroblast activation and vascular maturation occurring in a coordinated manner. These results suggest a potential cascade mechanism where M2 macrophage polarization coordinates with the moderate activation of TGF-β signaling to promote orderly collagen deposition, ultimately supporting functional ECM reconstruction with low-scarring tendency. In contrast, the Sal-CDs group displayed more diffuse TGF-β and α-SMA expression, suggesting that the tissue remained in a highly active proliferative stage. The PGA-DA and Control groups exhibited weaker signals, reflecting a comparatively delayed wound healing process.

Notably, in the early LPS-induced in vitro inflammatory model, PDS Gel suppressed the abnormal upregulation of TGF-β1 and α-SMA in fibroblasts, indicating its ability to inhibit excessive fibroblast activation under inflammatory conditions [61,62]. In contrast, during the late stages of in vivo wound healing, moderate upregulation of these molecules reflected the transition of the wound into the tissue remodeling phase. These findings suggest that PDS Gel may exert stage-specific regulatory effects: suppressing pathological activation under inflammatory conditions while supporting ECM reconstruction and tissue maturation during repair.

Additionally, bacterial colony counts in the wound area revealed that PDS Gel significantly reduced local *S. aureus* load, suggesting that the Sal-CDs component may provide antimicrobial activity in infected wounds, thereby stabilizing the local microenvironment for tissue repair (Fig. 9g and m).

Taken together, macroscopic healing observations, histological analysis, and immunostaining results indicate that PDS Gel can synergistically modulate inflammation, promote angiogenesis, and coordinate fibroblast-mediated matrix remodeling in infected wounds, leading to a more organized and stable tissue regeneration process.

The experimental data collectively demonstrate that PDS Gel successfully integrates multiple functions, including rapid wet-surface adhesion, ROS scavenging, immune modulation, and promotion of repair. The material exhibited significant efficacy in both the moist, immune-challenged oral ulcer model and the dry, infection-prone full-thickness skin defect model, strongly supporting its potential for cross-tissue repair. Despite the significant differences in the microenvironments of oral mucosa and skin (e.g., the contrast between salivary flow and dry environments), both share a common pathological bottleneck—persistent inflammation and oxidative stress. PDS Gel effectively accelerates wound healing across different tissues by directly targeting this bottleneck. Additionally, the skin model further validated the hydrogel's stable bioactivity and antimicrobial activity under infection conditions. Therefore, the PDS platform provides a universal and effective strategy for treating various epithelial tissue wounds obstructed by inflammation-related repair failure.

### 3.10 Limitations

To provide a balanced perspective, several limitations should be acknowledged. First, at the material-performance level, the effective adhesion window of the PDS Gel in the dynamic oral environment was approximately 1.5–2 h. While this duration is sufficient for the release and local bioactivity of the therapeutic agents following each administration, it may be insufficient for severe or chronic ulcers requiring prolonged drug coverage. Previous studies have reported sprayable rapid-gelling powders maintaining mucosal adhesion for over 12 h [63] and bilayer mucoadhesive patches exceeding 6 h [64], indicating room for improvement. The self-oxidative crosslinking strategy endowed the material with excellent sprayability and rapid in situ gelation, but also resulted in a relatively soft network (G′ and G″ < 1000 Pa). Such mechanical limitations are common in polysaccharide-based hydrogels [65,66] and, although acceptable for non-load-bearing oral mucosa, may restrict performance under higher shear or friction.

In addition, the acetic acid–induced recurrent ulcer model reproduces ulcer morphology and healing kinetics but primarily reflects chemical injury rather than the immune-driven pathophysiology of RAU. Clinical RAU involves T-cell–mediated cytotoxicity, inflammatory infiltration, cytokine imbalance (e.g., TNF-α, IL-2, IL-4), and genetic susceptibility [[67], [68], [69]], features that are difficult to capture in this model. Emerging immune-related models based on immune stimulation or autoantigen approaches aim to better mimic these mechanisms [70], although they are not yet standardized.

These limitations suggest directions for further study. Enhancing dopamine grafting density, constructing double/interpenetrating networks, or introducing dynamic covalent bonds may improve cohesion and retention stability [71]. Moreover, validation in immune-relevant ulcer models will be important to more comprehensively assess performance under T-cell–mediated inflammatory conditions and advance self-oxidatively crosslinked hydrogels toward realistic clinical scenarios.

## 4 Conclusion

In summary, we developed a multifunctional hydrogel (PDS Gel) based on PGA-DA and Sal-CDs using a “covalent crosslinking first, followed by self-oxidative physical crosslinking” strategy. The hydrogel combines sprayability with strong mucosal adhesion, ensuring firm wet adhesion immediately after application and effective wound support. Integration of Sal-CDs and a PDA-associated network confers antibacterial, ROS-scavenging, antioxidant, and immunomodulatory functions.

In vitro, PDS Gel modulated inflammation-related cellular behaviors, suppressed aberrant activation under inflammatory conditions, and enhanced endothelial angiogenic function and fibroblast-mediated ECM remodeling. Transcriptomic and immunological analyses further demonstrated that PDS Gel reprograms the immune microenvironment by regulating macrophage polarization and inflammatory signaling, favoring tissue repair. Together, these data suggest that ROS scavenging by PDS Gel may facilitate M2 macrophage polarization and downregulation of pro-inflammatory transcriptomic signatures, thereby contributing to the functional recovery of endothelial cells and fibroblasts, and is associated with organized collagen deposition and accelerated wound closure. In vivo, PDS Gel accelerated wound closure in both skin and oral ulcer models, while promoting epithelial regeneration, vascular maturation, and orderly ECM reconstruction, yielding superior tissue repair quality.

Collectively, this work presents a sprayable, adhesive hydrogel platform with structural robustness and multifunctional bioactivity, capable of rapidly alleviating inflammation and initiating tissue repair cascades. It provides a design blueprint for wound repair in complex dynamic environments and demonstrates strong potential for clinical translation.

## CRediT authorship contribution statement

**Lihong Zhou:** Conceptualization, Writing – original draft. **Jingyu Yan:** Investigation, Methodology. **Yurong Xu:** Investigation, Methodology. **Chenying Cui:** Investigation, Resources. **Kaifang Zhang:** Data curation, Formal analysis. **Kun Liu:** Investigation, Resources. **Xiuping Wu:** Funding acquisition, Project administration. **Bing Li:** Funding acquisition, Project administration.

## Declaration of competing interest

The authors declare that they have no known competing financial interests or personal relationships that could have appeared to influence the work reported in this paper.

## Data Availability

Data will be made available on request.
