# Supplementary material for: Self-oxidatively crosslinked sprayable hydrogel for microenvironment remodeling and accelerated healing of recurrent aphthous ulcers
Source: Mater Today Bio. 2026 May 14;38:103240. doi: 10.1016/j.mtbio.2026.103240 (PMC13214543; doi:10.1016/j.mtbio.2026.103240)
Supplement: Multimedia component 1 [file mmc1.docx]

**Supporting Information**

**Figures**


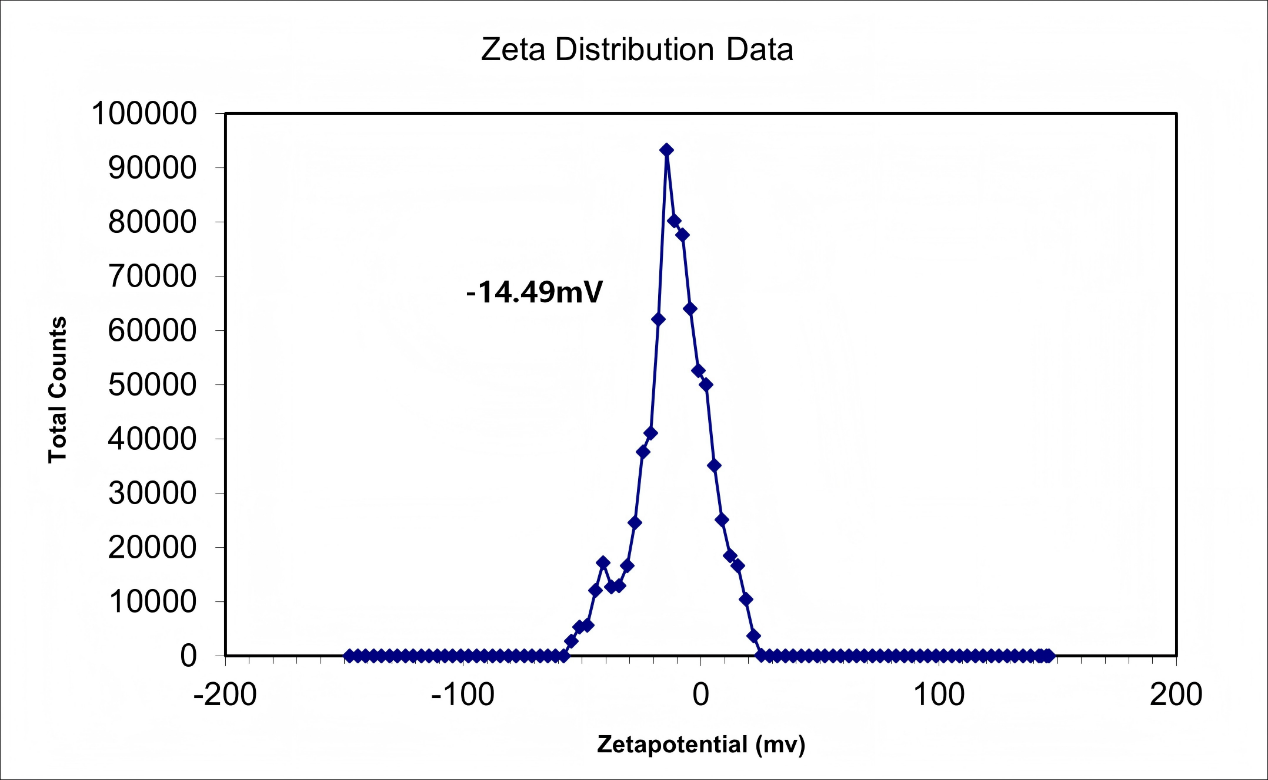


**Fig. S1.** Zeta potential of Sal-CDs.


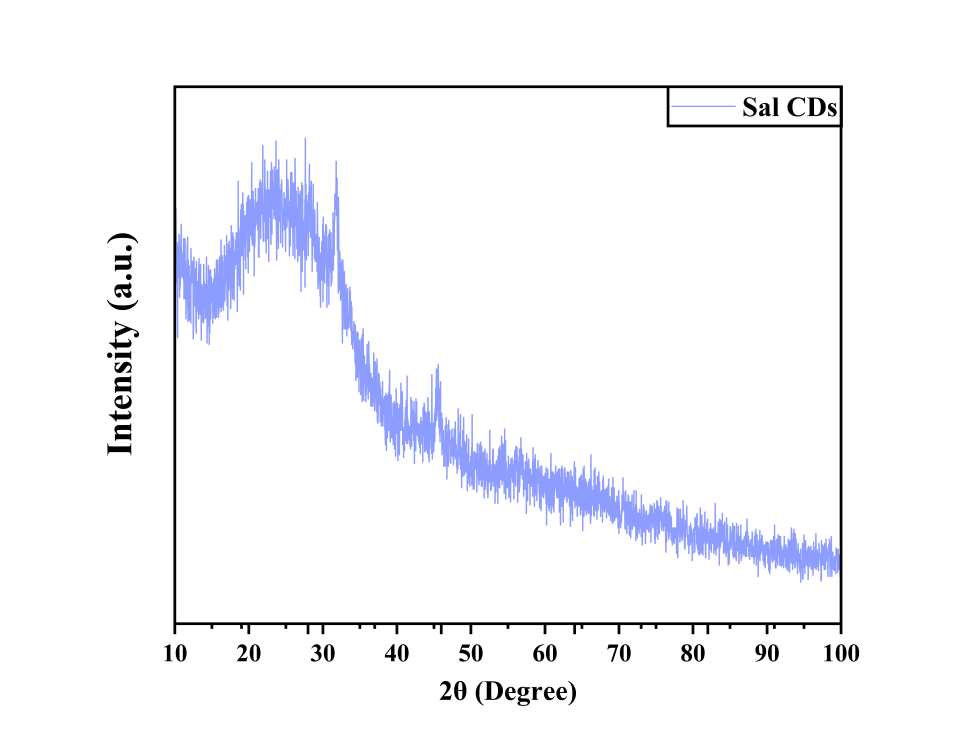


**Fig. S2.** XRD pattern of Sal-CDs.


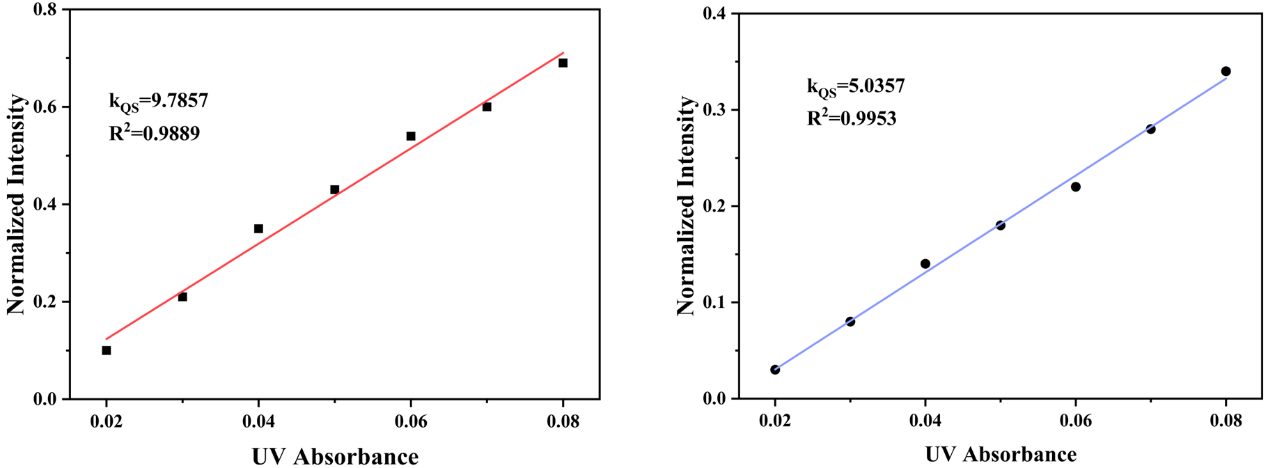


**Fig. S3.** The normalized intensity as a function of the UV-Vis absorbance with: (a) quinine sulfate as a reference and (b) the Sal-CDs as target.


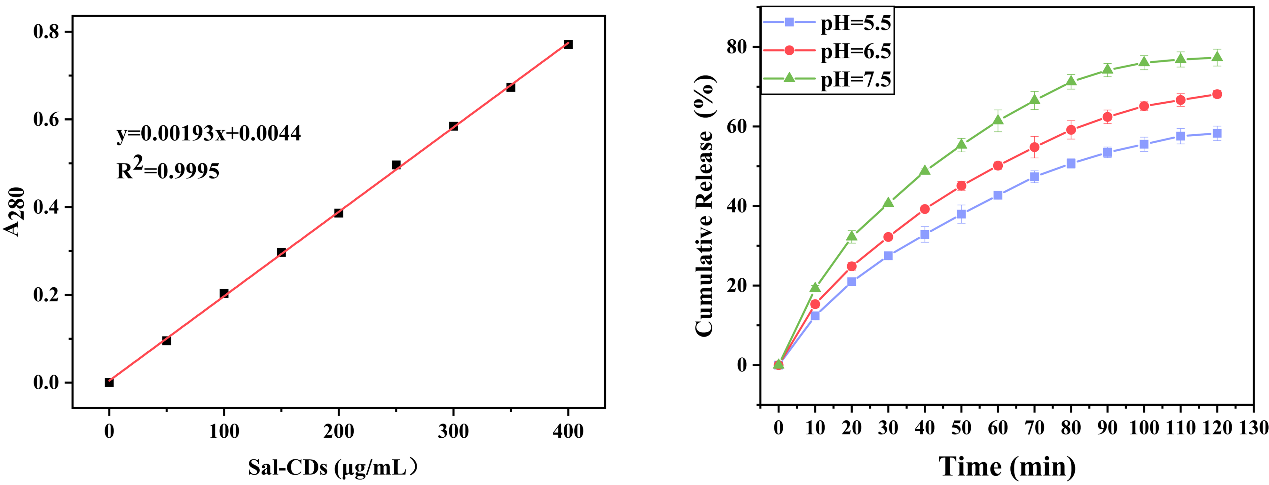


**Fig. S4.** Concentration–UV absorbance standard curve and release profiles of Sal-CDs at different pH values.


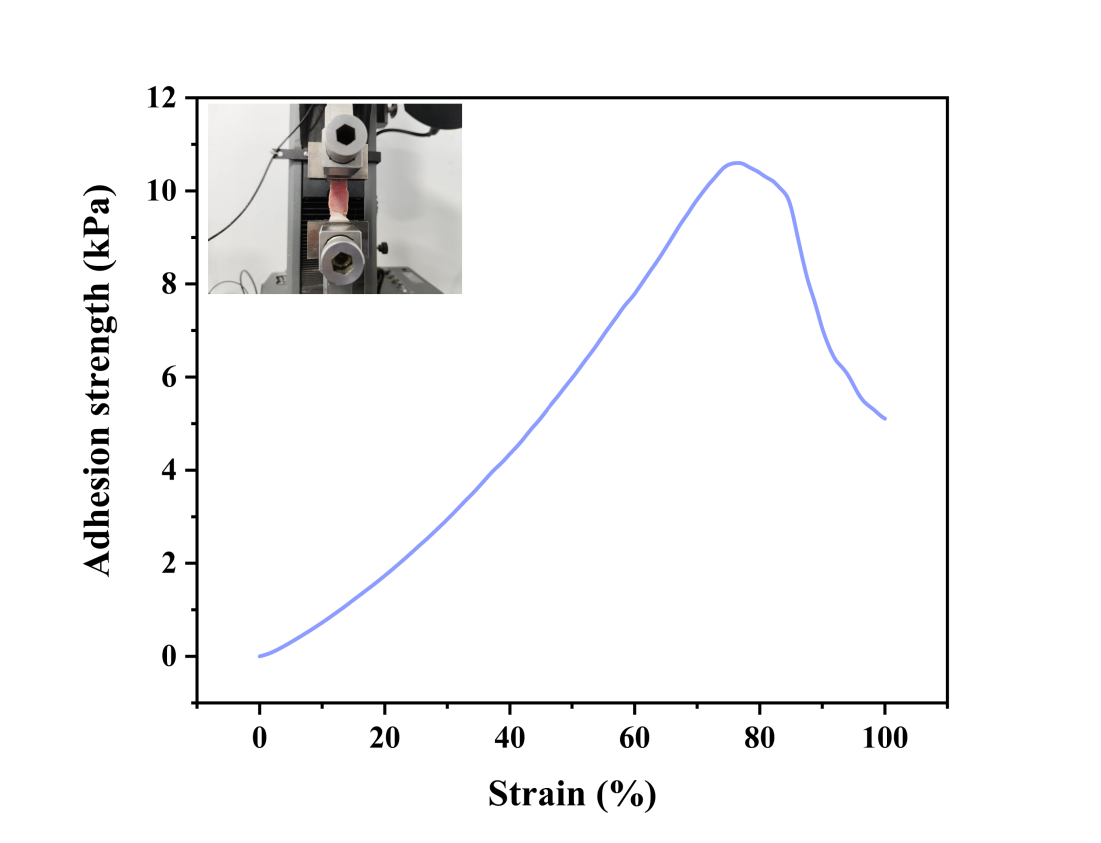


**Fig. S5.** Stress–strain curve of PDS Gel in the lap shear test on porcine oral mucosa.


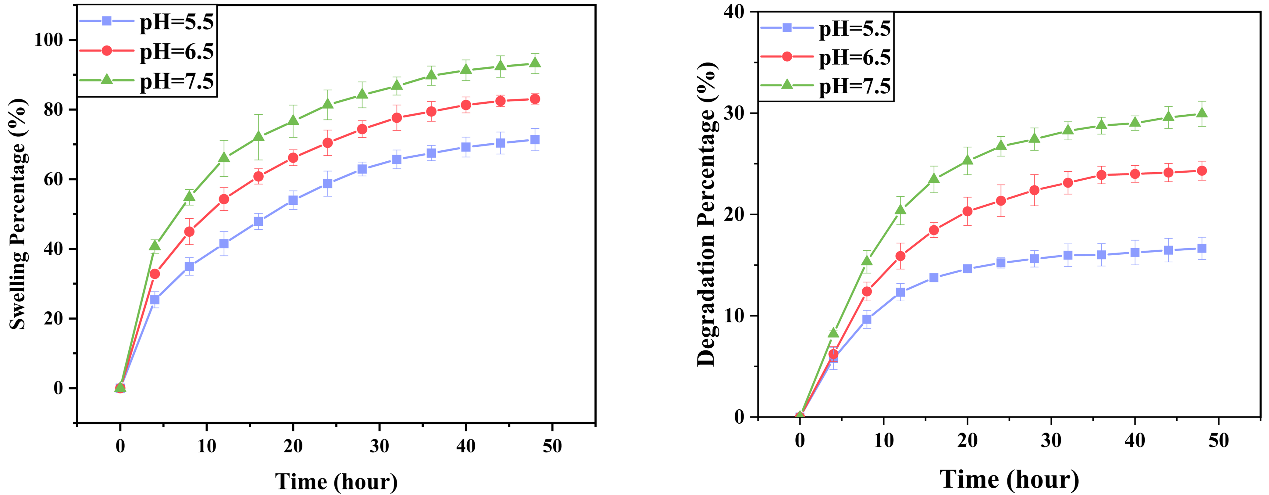


**Fig. S6.** Swelling curves (left) and degradation curves (right) of PDS Gel at different pH values.
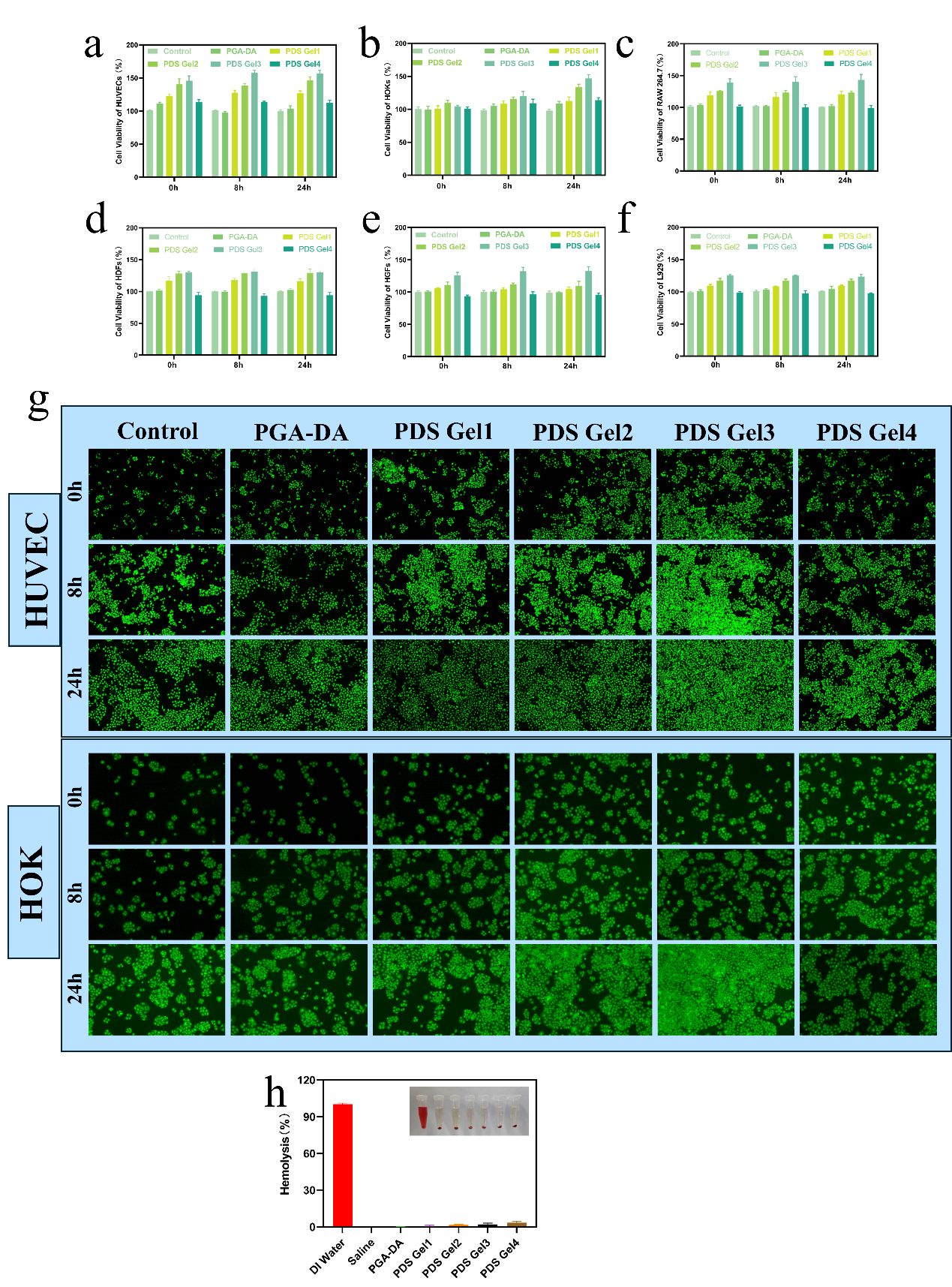


**Fig. S7.** In vitro biosafety evaluation of PDS Gel. Cell viability of HUVECs (a), HOKs (b), RAW264.7 cells (c), HDFs (d), HGFs (e), and L929 cells (f) cultured with hydrogels containing different concentrations of Sal-CDs, determined by the CCK-8 assay. (g) Cell proliferation of HUVECs and HOKs under different treatments at various time points, observed by Calcein AM staining (green). (h) Hemolysis evaluation of different material treatment groups.


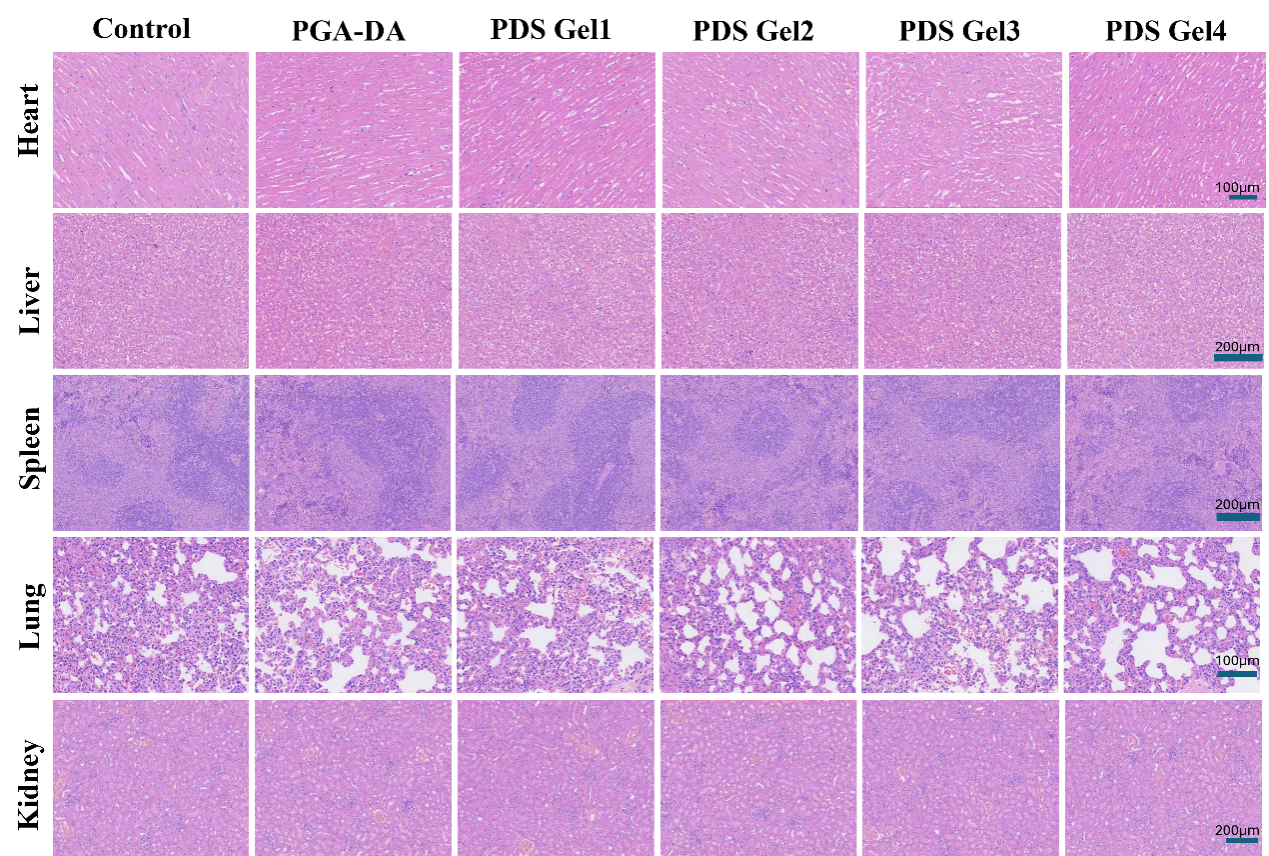


**Fig. S8.** H&E staining of major organs (heart, liver, spleen, lung, and kidney) after different treatments.


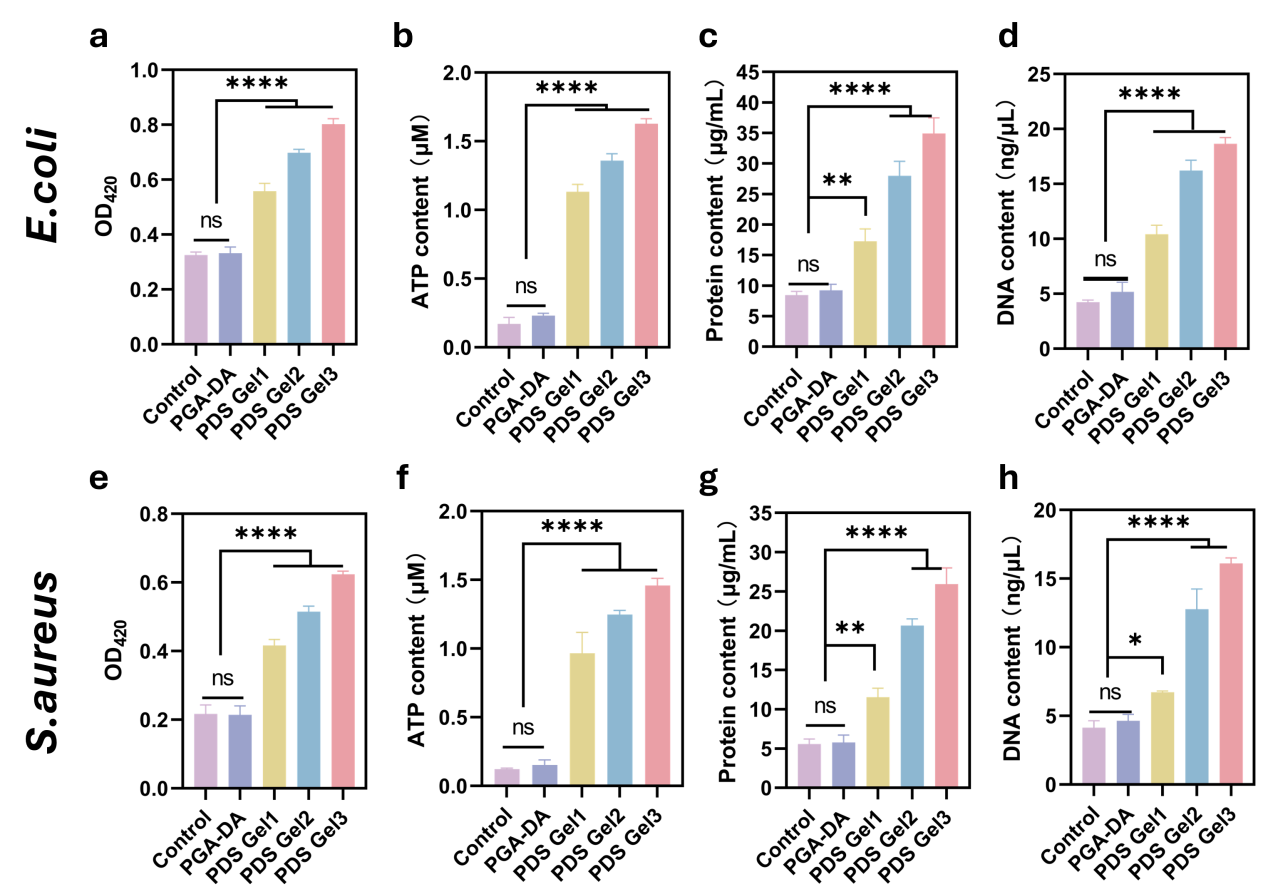


**Fig. S9.** Quantification of β-galactosidase (a, e), ATP (b, f), protein (c, g), and DNA (d, h) leakage from *E. coli* and *S. aureus* after treatment with different groups.


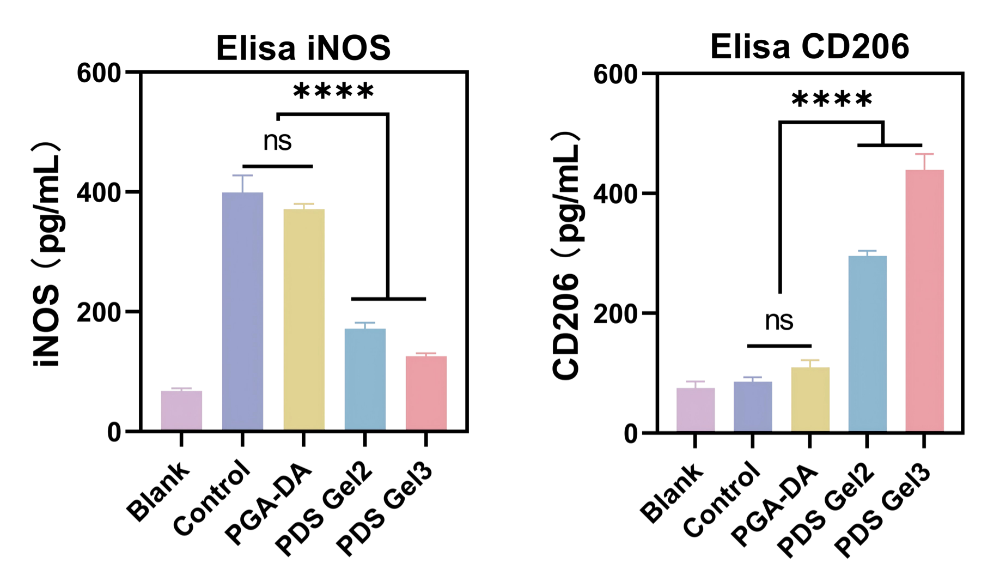


**Fig. S10.** ELISA quantification of iNOS and CD206 in macrophages.


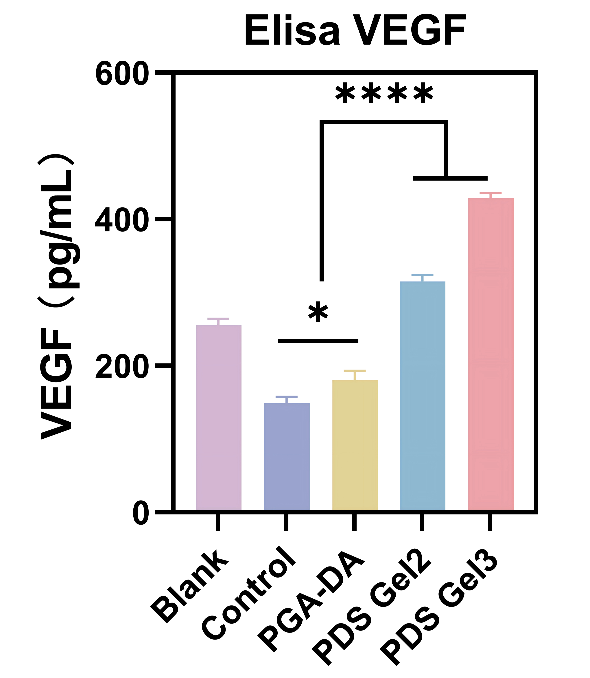


**Fig. S11.** ELISA quantification of VEGF secretion in HUVECs.


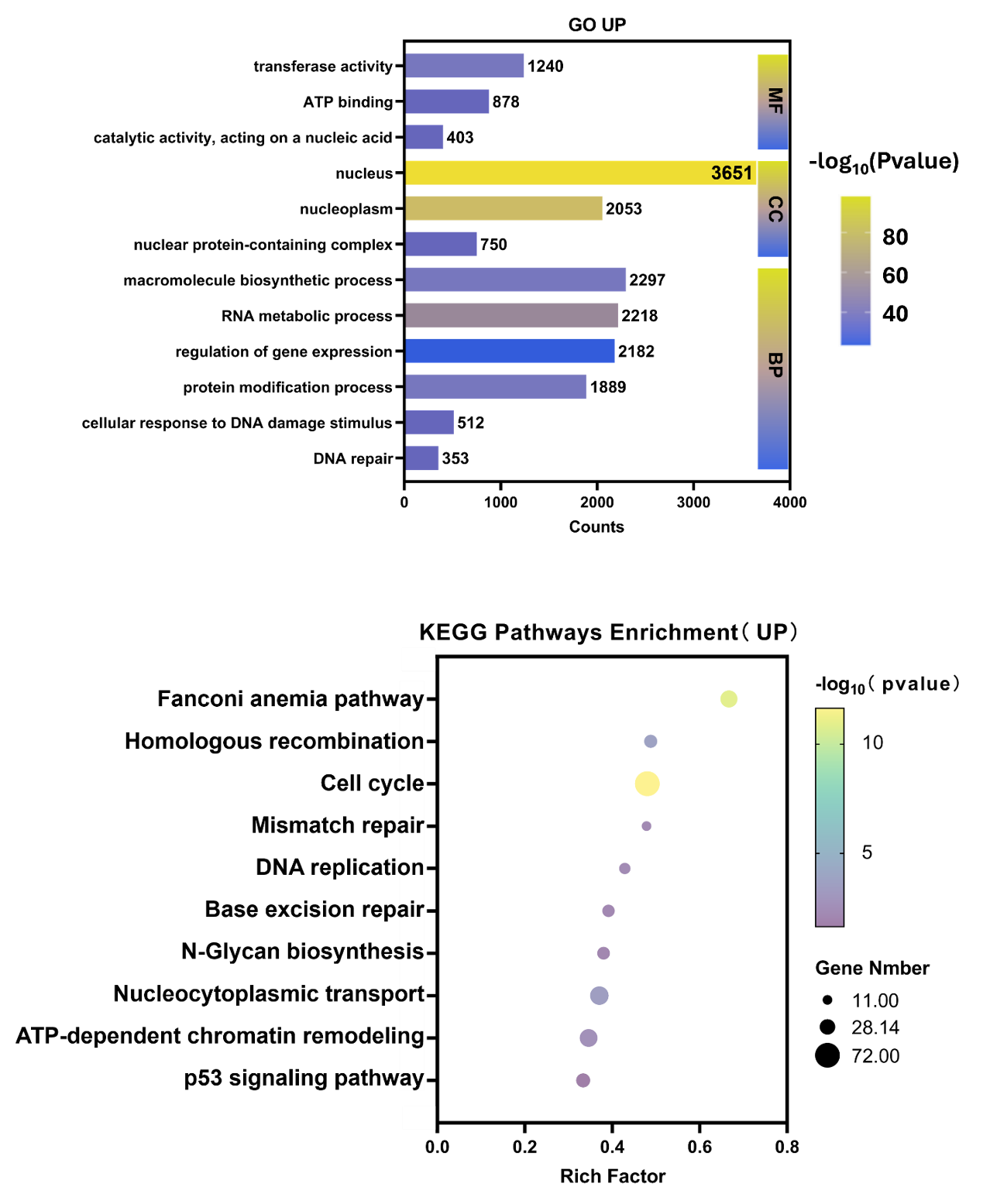


**Fig. S12.** Enrichment analysis of upregulated genes. (a) GO enrichment analysis showing significantly upregulated pathways. (b) KEGG enrichment analysis showing significantly upregulated pathways.


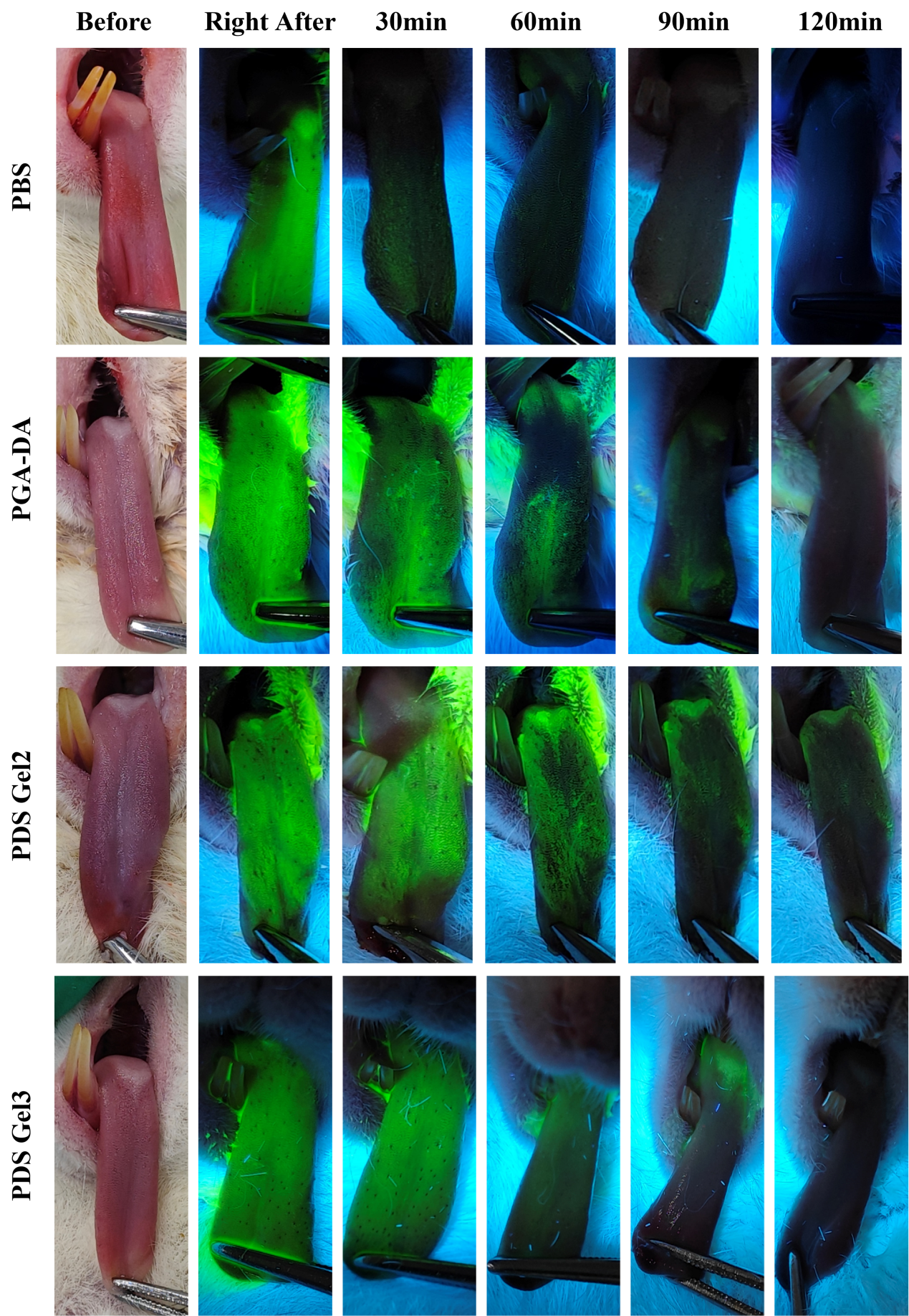


**Fig. S13.** Adhesion retention time test of PDS Gel on rat oral mucosa.


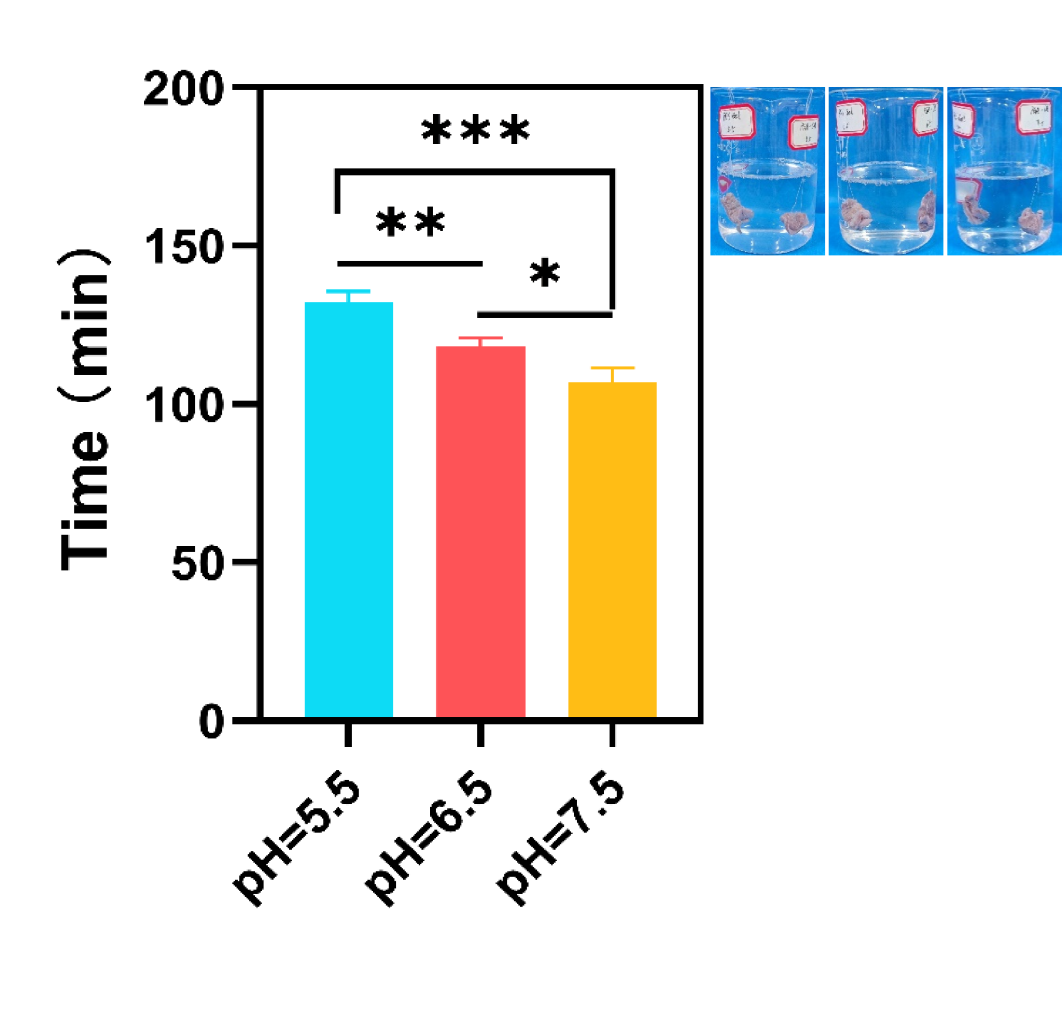


**Fig. S14.** Adhesion duration test on porcine oral mucosa.

**
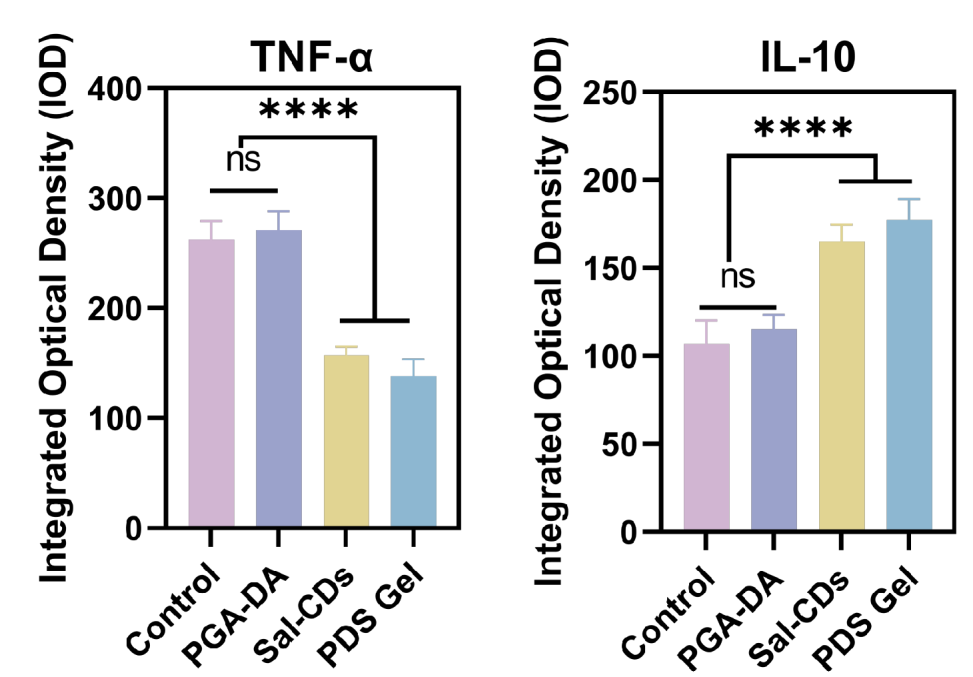
**

**Fig. S15.** Immunohistochemical quantification of TNF-α and IL-10 in oral ulcer wounds.


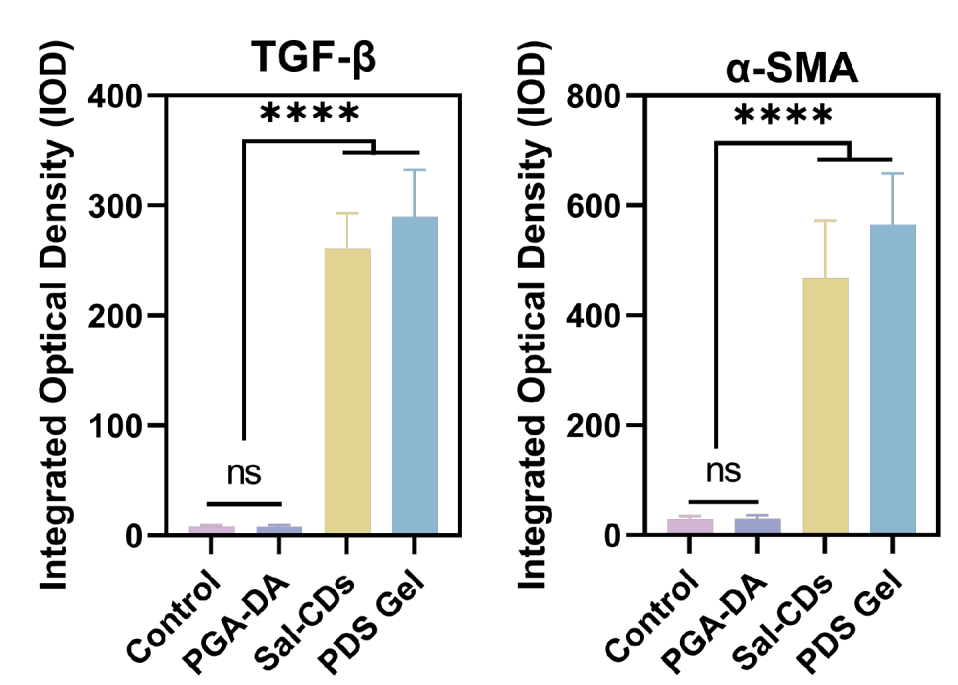


**Fig. S16.** Immunohistochemical quantification of TGF-β and α-SMA in skin defect wounds.

**Table**

**Table S1.** Primers used in the qRT-PCR analysis.

| Genes | Forward primer (5’-3’) | Reverse primer (5’-3’) |
| --- | --- | --- |
| TNF-*α* | CTTGTTGCCTCCTCTTTTGCTTA | CTTTATTTCTCTCAATGACCCGTAG |
| IL-1*β* | TGTGTTTTCCTCCTTGCCTCTGAT | TGCTGCCTAATGTCCCCTTGAAT |
| IL-6 | ATA ACC ACC CCT GAC CCA AC | CCC ATG CTA CAT TTG CCG AA |
| IL-10 | GGAAGACAATAACTGCACCCACT | CAACCCAAGTAACCCTTAAAGTCC |
| Arg-1 | CCCAGCTTGTCTACTTCAGTCATG | GGCAACCTGTGTCCTTTCTCCT |
| VEGF | GTCCTCTCCTTACCCCACCTCCT | CTCACACACACAGCCAAGTCTCCT |
| α-SMA | GACAATGGCTCTGGGCTCTGTAA | TGTGCTTCGTCACCCACGTA |
| COL1A1 | GATTCCCTGGACCTAAAGGTGC | AGCCTCTCCATCTTTGCCAGCA |
| FN1 | ACAACACCGAGGTGACTGAGAC | GGACACAACGATGCTTCCTGAG |
| Nos2 | TGAAGAAAACCCCTTGTGCT | TTCTGTGCTGTCCCAGTGAG |
| Mrc1 | GGAA TCAAGGGCACAGAGTTA | ATTGTGGAGCAGATGGAA |
| TGF-β | CACCCGCGTGCTAATGG | ATGCGTGTGTACTCTGCTTGAACT |
| GAPDH | AGAAGGTGGTGAAGCAGGCATC | CGAAGGTGGAAGAGTGGGAGTTG |
